# Orthogonal chemical genomics approaches reveal genomic targets for increasing anaerobic chemical tolerance in *Zymomonas mobilis*

**DOI:** 10.1128/msystems.01001-25

**Published:** 2025-12-04

**Authors:** Jacob B. Eckmann, Amy L. Enright Steinberger, Morgan Davies, Elizabeth Whelan, Kevin S. Myers, Margaret L. Robinson, Amy B. Banta, Piyush B. Lal, Joshua J. Coon, Trey K. Sato, Patricia J. Kiley, Jason M. Peters

**Affiliations:** 1 DOE Great Lakes Bioenergy Research Center, University of Wisconsin-Madison 5228, Madison, Wisconsin, USA; 2 Department of Biomolecular Chemistry, University of Wisconsin-Madison 5228, Madison, Wisconsin, USA; 3 Pharmaceutical Sciences Division, School of Pharmacy, University of Wisconsin-Madison 5228, Madison, Wisconsin, USA; 4 Microbiology Doctoral Training Program, University of Wisconsin-Madison 5228, Madison, Wisconsin, USA; 5 Morgridge Institute for Research 145254, Madison, Wisconsin, USA; 6 Department of Chemistry, University of Wisconsin-Madison 5228, Madison, Wisconsin, USA; 7 National Center for Quantitative Biology of Complex Systems, University of Wisconsin-Madison 5228, Madison, Wisconsin, USA; 8 Department of Bacteriology, University of Wisconsin-Madison 5228, Madison, Wisconsin, USA; 9 Department of Medical Microbiology and Immunology, University of Wisconsin-Madison 5228, Madison, Wisconsin, USA; 10 Center for Genomic Science Innovation, University of Wisconsin-Madison 5228, Madison, Wisconsin, USA; Broad Institute, Cambridge, Massachusetts, USA

**Keywords:** TnSeq, CRISPRi, phenolic acids, electron transport chain, TonB-dependent receptors

## Abstract

Genetically engineered microbes have the potential to increase efficiency in the bioeconomy by overcoming growth-limiting production stress. Screens of gene perturbation libraries against production stressors can identify high-value engineering targets, but follow-up experiments needed to guard against false positives are slow and resource-intensive. In principle, the use of orthogonal gene perturbation approaches could increase recovery of true positives over false positives because the strengths of one technique compensate for the weaknesses of the other, but, in practice, two parallel screens are rarely performed at the genome scale. Here, we screen genome-scale CRISPRi (CRISPR interference) knockdown and transposon insertion libraries of the bioenergy-relevant Alphaproteobacterium, *Zymomonas mobilis*, against growth inhibitors commonly found in deconstructed plant material. Integrating data from the two gene perturbation techniques, we established an approach for defining engineering targets with high specificity. This allowed us to identify all known genes in the cytochrome *bc*_1_ and cytochrome *c* synthesis pathway as potential targets for engineering resistance to phenolic acids under anaerobic conditions, a subset of which we validated using precise gene deletions. Strikingly, this finding is specific to the cytochrome *bc*_1_ and cytochrome *c* pathway and does not extend to other branches of the electron transport chain. We further show that exposure of *Z. mobilis* to ferulic acid causes substantial remodeling of the cell envelope proteome, as well as the downregulation of TonB-dependent transporters. Our work provides a generalizable strategy for identifying high-value engineering targets from gene perturbation screens that is broadly applicable.

**IMPORTANCE:**

Engineering microorganisms to tolerate harsh production conditions will contribute to increased bioproduct yields. In this study, we systematically identified *Zymomonas mobilis* genes that confer resistance or susceptibility to chemical stressors found in deconstructed plant material. We used complementary genetic techniques to cross-validate these genes at scale, providing a widely applicable method for precisely identifying genetic alterations that increase chemical resilience. We discovered genetic modifications that improve anaerobic growth of *Z. mobilis* in the presence of inhibitory chemicals found in renewable plant-based feedstocks. These results have implications for engineering robust production strains to support efficient and resilient bioproduction. Our methodologies can be broadly applied to understand microbial responses to chemicals across systems, paving the way for developments in biomanufacturing, therapeutics, and agriculture.

## INTRODUCTION

Industrial-scale fermentation by genetically engineered microorganisms can produce a wide array of products, including fuels and chemicals traditionally derived from petroleum. Over the last several decades, renewable plant biomass has emerged as a promising carbon source for conversion to value-added bioproducts (1, 2). However, plant-based feedstocks also contain a myriad of known and unknown chemical stressors that slow microbial growth and limit fermentation yields (3–5). These chemicals are derived from the breakdown of lignocellulose from plant cell walls (e.g., aromatic and phenolic acids, furans), solvents used to unlock sugars from biomass (e.g., ammonia or γ-valerolactone) or fermentation products themselves (e.g., ethanol and isobutanol) and can affect microorganisms through a broad array of mechanisms, including changing membrane permeability and intracellular pH, generating reactive oxygen species, interacting with and mutagenizing DNA, and directly inhibiting cellular enzymes (5–11). Engineering microorganisms to better tolerate these chemical stressors stands to increase fermentation yields.

One way to identify genetic engineering targets that improve resilience is through chemical genomics, where mutant libraries are screened against a suite of inhibitory chemicals to reveal changes in mutant fitness resulting from chemical exposure (12–17). As a high-throughput assay, chemical genomics is subject to false positives that waste time and resources to follow up if not avoided. Parallel use of multiple orthogonal mutant libraries could theoretically reduce false positives through cross-validation of phenotypes, though this approach is underexplored.

Random transposon (Tn) and CRISPR interference (CRISPRi) libraries are ideal tools for this comparative approach, as they have complementary strengths and weaknesses (18–20). For example, screening Tn libraries can identify essential genes but cannot further characterize their chemical phenotypes because transposon insertion in an essential gene is lethal by definition. CRISPRi libraries can be designed to overcome this limitation by including mismatched single guide RNAs (sgRNAs) for partial knockdown of essential genes (Mismatch-CRISPRi) or by partially inducing CRISPRi components, maintaining cell viability and allowing for phenotype identification (21, 22). Additionally, the two technologies cause different *cis* effects on downstream gene expression, potentially leading to distinct types of false positives. CRISPRi reliably reduces expression of the gene target and all downstream genes in a transcription unit, a phenomenon called polarity (23, 24). Conversely, transposons often contain a strong promoter to drive expression of a selective marker, and transcription across the insertion junction can result in readthrough expression of downstream genes. This has been used as a strategy for deliberate screening of gene overexpression phenotypes (25). Integration of both approaches could reduce false positives by identifying targets with consistent phenotypes despite differing *cis* effects.

The Alphaproteobacterium *Zymomonas mobilis*, a facultative anaerobe well known for its native ability to rapidly convert sugars into ethanol, is a promising organism for industrial fermentation of renewable plant biomass to bioproducts (26–29). Enhancing its industrial appeal, *Z. mobilis* features high native tolerance to ethanol and some, but not all, lignocellulose-derived chemicals (30, 31). It also features a small genome with an improving toolkit for genomic manipulation (31–47). However, much of the *Z. mobilis* genome remains unannotated, impeding rational engineering efforts to further enhance stress tolerance. To address this challenge, other groups have performed chemical genomics on *Z. mobilis* Tn libraries grown aerobically in bioenergy-relevant inhibitors using a microarray-based quantification approach that was a predecessor to Tn insertion sequencing (Tn-seq) (15, 48). However, large batch industrial fermentations with *Z. mobilis* are likely to be performed anaerobically because its optimal growth environment is anaerobic and aerating industrial-scale fermenters is costly. Thus, deciphering how *Z. mobilis* responds to inhibitory production stressors under anaerobic conditions remains an important question.

In this study, we use Tn insertion and CRISPRi as orthogonal approaches to identify genes that alter susceptibility to fermentation-relevant chemicals in *Z. mobilis* under anaerobic conditions. We quantitatively compare the two techniques to increase the reliability of discoveries by enriching for true positives. Our screens reveal a surprising role for an electron transport pathway in anaerobic chemical stress caused by phenolic acids (e.g., ferulic acid) encountered during microbial fermentation of plant biomass, providing genetic engineering targets for increasing microbial growth and bioproduct yields. Finally, we characterize the effect of ferulic acid on the *Z. mobilis* proteome, revealing potential mechanisms of phenolic acid-induced stress.

## RESULTS

### Parallel screening detects genes that alter fitness against production stressors

To identify *Z. mobilis* genes that modulate fitness in the presence of industrially relevant chemicals, we screened Tn and CRISPRi libraries anaerobically against a suite of inhibitory production stressors, which represent the various classes of fermentation-relevant chemicals, including phenolic acids, furans, solvents, and fermentation end products (Fig. S1; Table S1). Libraries were grown at a range of sub-lethal concentrations for each chemical. For each gene knockout (Tn) or knockdown (CRISPRi), we calculated a chemical-gene (CG) score representing the log_2_ fold change in relative mutant abundance between chemical-treated and untreated cultures. Positive CG scores indicate mutants that have increased fitness with the chemical, while negative CG scores indicate mutants that have decreased fitness.

**Fig 1:**
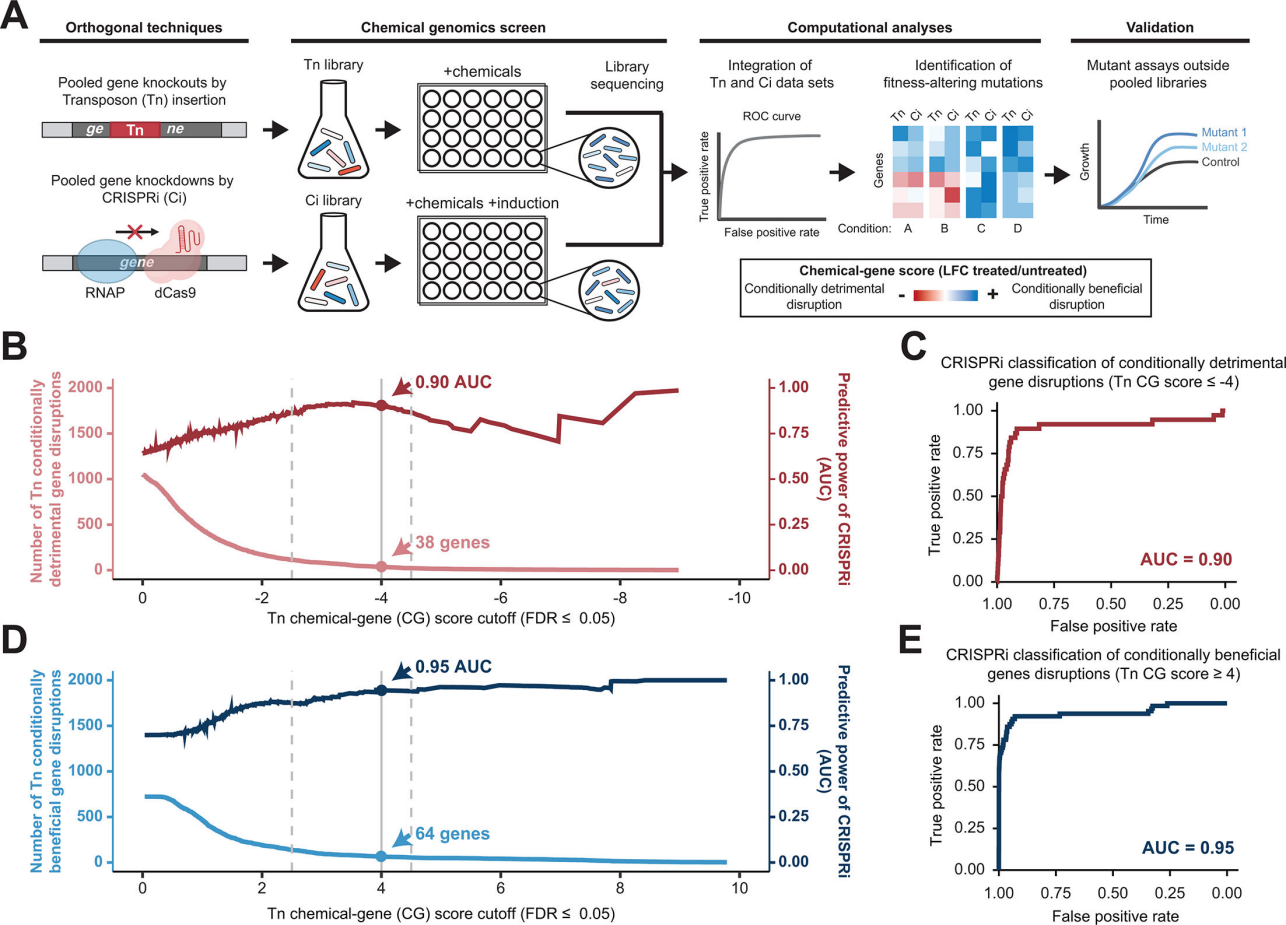
Parallel *Z. mobilis* chemical genomics screens using orthogonal Tn and CRISPRi libraries. (**A**) Schematic of integrative chemical genomics using Tn and CRISPRi libraries grown with fermentation-relevant chemicals. RNAP, RNA polymerase. (**B and D**) Receiver operating characteristic (ROC) curve area under the curve (AUC) across CG score cutoff values for conditionally detrimental (**B**) and conditionally beneficial (**D**) gene disruptions, using the Tn library as ground truth. The darker line depicts AUC at a given CG score cutoff (right *y*-axis) while the lighter line represents the number of genes that pass the associated cutoff value (left *y*-axis). Dashed vertical lines approximate the bounds of a flexible range within which researchers may choose to select cutoffs by weighing the tradeoff between the number of hits and the reliability of those hits. The solid gray line at |CG score| = 4 marks the score cutoff chosen in this work to identify genes for further study. (**C and E**) ROC curves generated using (**C**) CG score ≤−4 and (**E**) CG score ≥4 using the Tn library as ground truth.

Theorizing that parallel use of orthogonal techniques could increase reliability of results, we developed a method for cross-validating findings between the two libraries (Fig. 1A). Various technical factors, such as sequencing depth, library size, and population doublings, may impact the spread of CG scores for each library in each condition. To account for these factors, we first quantile-normalized the CG scores for non-essential genes (i.e., genes that could be assessed by both the Tn and CRISPRi libraries) for both libraries within each condition, producing consistent distributions of CG scores that facilitate direct comparison of the data sets (Fig. S2).

**Fig 2:**
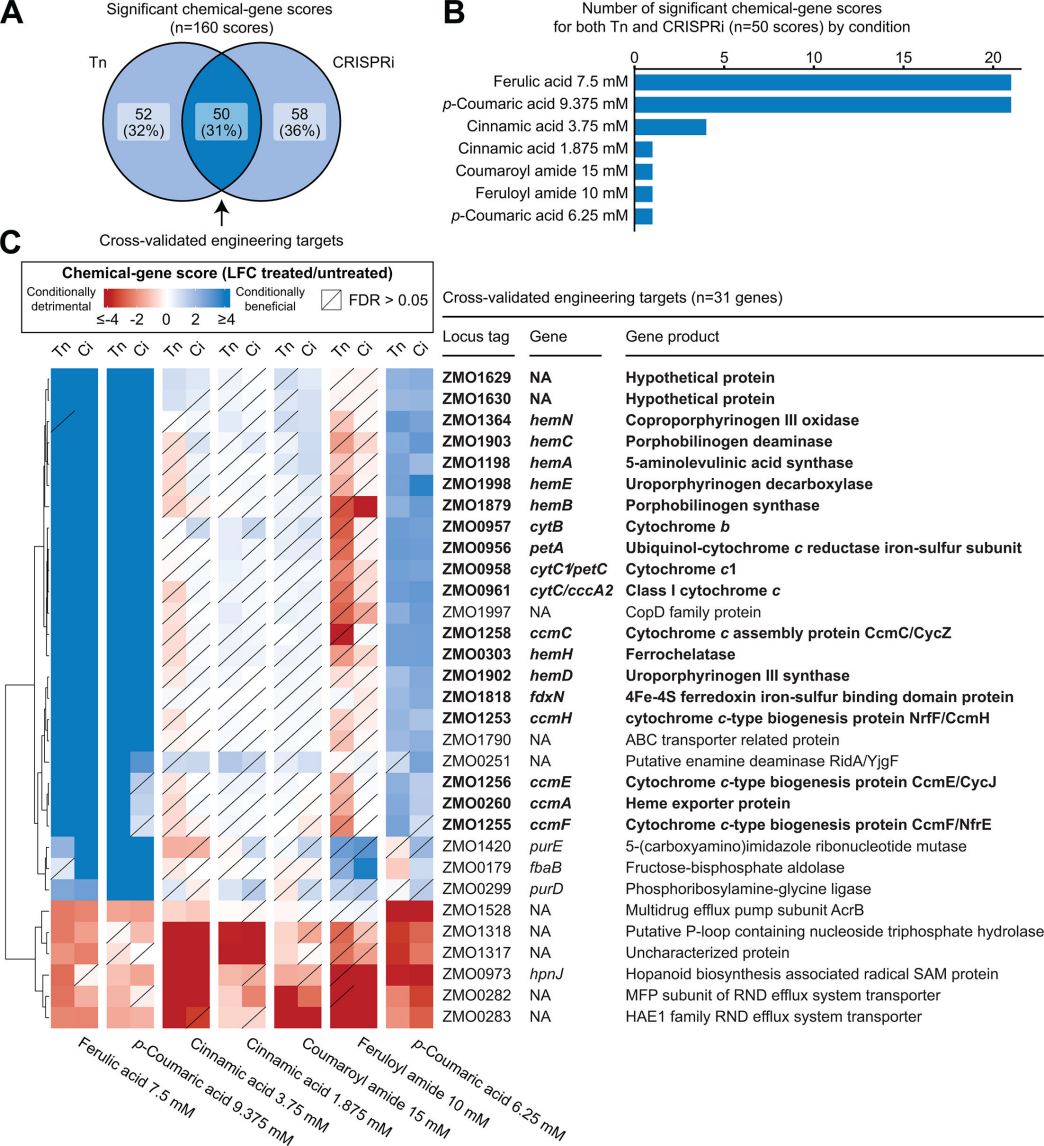
Significant chemical genomics hits identified by Tn and CRISPRi libraries. (**A**) Venn diagram showing the number of significant phenotypes identified by either Tn or CRISPRi, or in both. (**B**) Chart depicting the number of significant genes (|chemical-gene score| ≥ 4 in both Tn and CRISPRi) in each condition that had at least one significant gene. (**C**) Hierarchical clustered heatmap showing chemical-gene scores for significant genes across conditions that had at least one significant gene. Bolded genes are also displayed in Fig. 3A. Tn, transposon library; Ci, CRISPRi library. Diagonal lines indicate statistical non-significance (FDR > 0.05). Hierarchical clustering was applied only to the *y*-axis (gene axis). Conditions are arranged from left to right by the number of significant genes, as displayed in panel **A**.

Next, we systematically determined a CG score cutoff to apply across libraries and reliably define engineering targets. To do so, we constructed receiver operating characteristic (ROC) curves that test the ability of one library to accurately predict results from the other, where a higher area under the curve (AUC) represents greater prediction accuracy. This strategy was previously used to evaluate the ability of an *Escherichia coli* CRISPRi library to classify known essential genes based on gold-standard deletion libraries (49). For each of our libraries, we designated gene disruptions as either conditionally detrimental (disruption decreases fitness; negative CG scores) (Fig. 1B and C) or conditionally beneficial (disruption increases fitness; positive CG scores) (Fig. 1D and E) along a range of CG score cutoffs (and false discovery rate [FDR] ≤ 0.05) and calculated AUC for ROC curves at each cutoff. As the cutoff increases, the number of genes passing a given cutoff decreases while predictive power generally increases until the gene sample size becomes insufficient, causing noise in the AUC calculation (e.g., Fig. 1B). Therefore, there is a tradeoff between the number of genes identified and the reliable classification of gene phenotypes, and researchers may opt for more stringent or relaxed cutoffs depending on their tolerance for false results and bandwidth for follow-up experiments. For this study, we chose a cutoff of |CG score| ≥4. At this cutoff, CRISPRi library CG scores reliably classify Tn mutant phenotypes (AUC = 0.90 for 38 conditionally detrimental gene disruptions and AUC = 0.95 for 64 conditionally beneficial gene disruptions) (Fig. 1B and D; Fig. S3). In the same manner, Tn CG scores are able to reliably classify CRISPRi mutant phenotypes at this cutoff (AUC = 0.86 for 43 conditionally detrimental gene disruptions and AUC = 0.94 for 65 conditionally beneficial gene disruptions) (Fig. S3). Applying this cutoff, we identified 103 genes with significant CG scores (|CG score| ≥ 4, FDR ≤ 0.05) in at least one chemical condition and genetic library (Fig. S3, Tables S2 and S3). Of the 103 significant genes, 31 had significant CG scores in both libraries for at least one condition (Fig. 2A). We consider these genes with cross-validated phenotypes to be potential engineering targets for generating inhibitor-resistant production strains. The other 72 genes were significant in one library but not in the other and may warrant further investigation (Table S3 and Fig. S4).

### Disruption of the cyt *bc***_1_**/cyt *c* electron transport pathway increases fitness in phenolic acids

Of the 31 cross-validated engineering targets, 21 gene disruptions improved fitness in ferulic acid and *p*-coumaric acid (Fig. 2B and C; Fig. S1). Strikingly, these 21 genes include all known structural genes needed to synthesize a quinol-oxidizing electron transport pathway consisting of a cytoplasmic membrane-bound cytochrome *bc*_1_ complex (*ZMO0956-ZMO958*) and a periplasmic cytochrome *c* (*ZMO0961*) (herein termed the cyt *bc*_1_/cyt *c* pathway). Notably, the electron transfer components that this pathway interacts with and any final electron acceptors are unknown. Other genes required for maturation of these respiratory enzymes had similarly positive CG scores in these conditions, including genes encoding synthesis of heme, an essential cofactor for cytochrome-mediated electron transfer (50–52), and the cytochrome *c* maturation (*ccm*) complex, required for covalent heme attachment to cytochromes *c* and translocation to the periplasm (53–55). Hierarchical clustering of CG scores across all conditions also revealed that the cytoplasmic ferredoxin *fdxN* (*ZMO1818*) and two members (*ZMO1629, ZMO1630*) of an operon of unknown function (*ZMO1631-ZMO1628*) clustered with cyt *bc*_1_/cyt *c* pathway genes (Fig. S4). While *ZMO1631* is annotated as a TonB-dependent receptor, its downstream genes (*ZMO1630-ZMO1628*) are poorly annotated. ZMO1630-1628 are predicted to have α-helices indicative of localization to the cytoplasmic membrane. Furthermore, AlphaFold3 modeling (56) predicts that ZMO1630-ZMO1628 form a transmembrane complex (ipTM = 0.86; Fig. S6) but are not predicted to associate with ZMO1631 (ipTM = 0.57). These uncharacterized genes were included in follow-up analyses due to their similar phenotypes, which suggest they have related functions. Importantly, genes encoding other quinol-oxidizing pathways, such as cytochrome *bd* oxidase (*ZMO1571-1572*) and cytochrome *c* peroxidase (*ZMO1136*), did not show significant phenotypes in any condition, indicating that the increased phenolic acid resistance phenotype is specific to the cyt *bc*_1_/cyt *c* pathway rather than electron transport pathways in general (Fig. S7). These fitness patterns were specific to phenolic acids and did not extend to non-phenolic cinnamic acid or the phenolic amides, feruloyl and coumaroyl amide (Fig. 2C).

**Fig 3:**
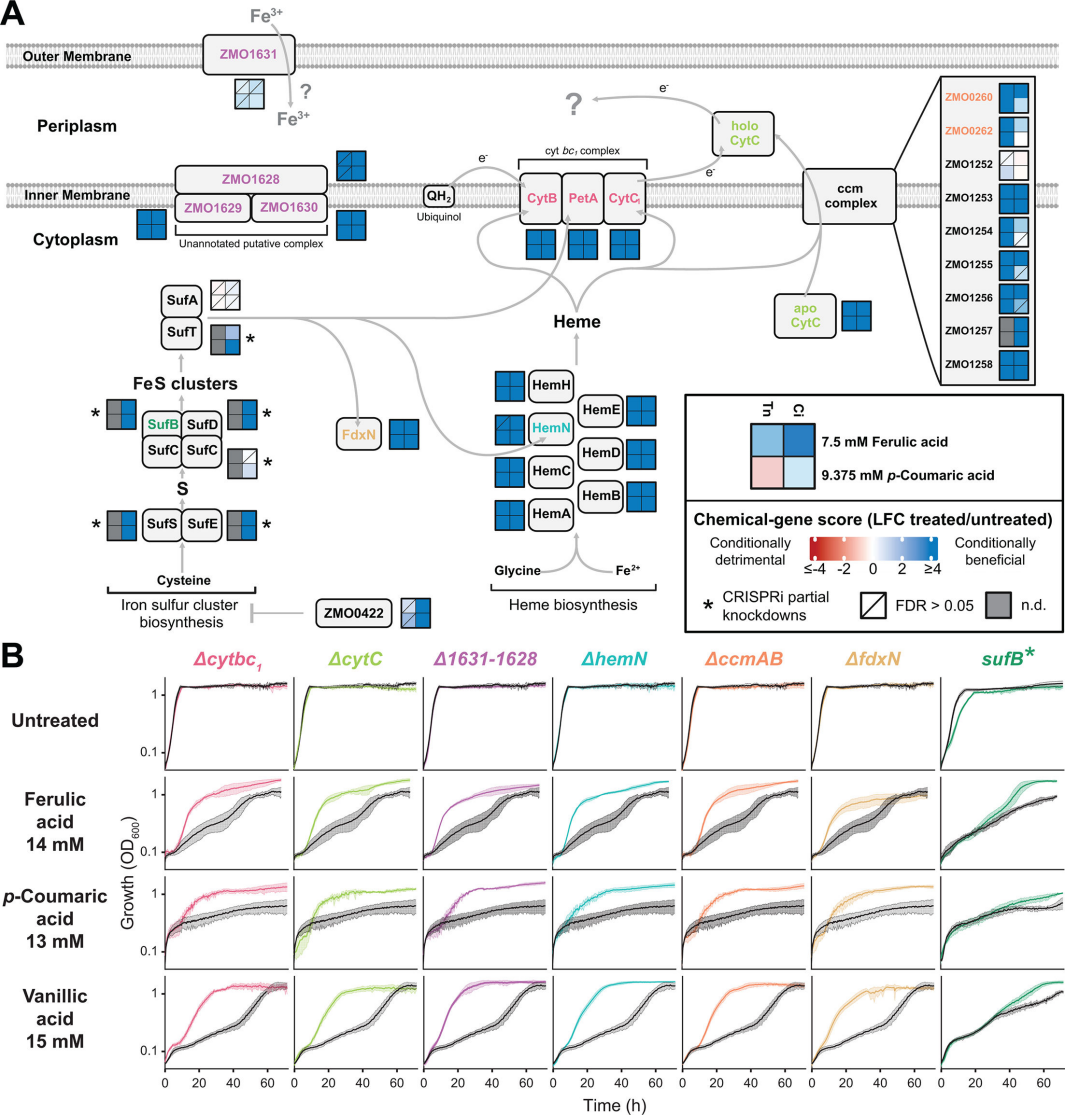
Fitness and growth of functionally related gene mutants in ferulic and *p*-coumaric acid. (**A**) Pathway map showing functional relationships between genes that had significant phenotypes in 7.5 mM ferulic and 9.375 mM *p*-coumaric acid. Heatmaps next to each gene depict chemical-gene scores in both conditions for Tn and CRISPRi libraries. Tn, transposon library; Ci, CRISPRi library; n.d., no data. Diagonal lines indicate statistical non-significance (FDR > 0.05). Heatmaps with asterisks display the median CG score for CRISPRi partial knockdown mutants using mismatched sgRNAs. n.d. denotes no data. Corresponding locus tags for protein names shown are SufB, ZMO0423; SufC, ZMO0425; SufD, ZMO0426; SufS, ZMO0427; SufT, ZMO0428; SufA, ZMO0429; SufE, ZMO1067; HemA, ZMO1198; HemB, ZMO1879; HemC, ZMO1903; HemD, ZMO1902; HemN, ZMO1364; HemE, ZMO1998; HemH, ZMO0303; CytB, ZMO0957; PetA, ZMO0956; CytC_1_, ZMO0958; CytC, ZMO0961; FdxN, ZMO1818. (**B**) Anaerobic growth curves with phenolic acids for deletion mutants or a *sufB* Mismatch-CRISPRi partial knockdown mutant (colored lines) and wild-type *Z. mobilis* or a non-targeting CRISPRi control strain, respectively (black lines). Growth curve error bars are standard deviations of quadruplicate samples.

Iron–sulfur clusters also play an indispensable role in the cyt *bc*_1_/cyt *c* pathway, with the Rieske iron–sulfur protein (*ZMO0956*) and *hemN* utilizing [2Fe–2S] and [4Fe–4S] clusters, respectively (39, 40). Additionally, the ferredoxin *fdxN* utilizes a [4Fe–4S] cluster (57). We therefore reasoned that disruption of Fe–S cluster biosynthesis should provide a similar fitness benefit with phenolic acids. However, genes encoding the iron–sulfur cluster biosynthetic Suf system (58) are essential and therefore are absent from our Tn and CRISPRi library comparisons since Tn insertion into essential *suf* genes is lethal (59 and this study). The notable exception to this is the first gene in the *suf* operon, *ZMO0422*, which encodes a non-essential predicted transcription factor (Fig. S8). While Tn mutants of *ZMO0422* showed minimal phenotypic effects, CRISPRi mutants exhibited strong positive scores in the presence of ferulic and *p*-coumaric acids, likely due to reduced transcription of the downstream *suf* genes. Consistent with this, partial knockdown of *suf* genes using mismatched sgRNAs in the CRISPRi library resulted in similarly positive phenotypes with ferulic and *p*-coumaric acids (Fig. 3A), confirming that hindering Fe–S cluster biosynthesis also increases fitness with these compounds, possibly by disrupting maturation of the cyt *bc*_1_/cyt *c* pathway.

We next validated that disruption of the cyt *bc*_1_/cyt *c* pathway improves fitness against phenolic acids in a non-competitive assay. We constructed in-frame deletions to generate null mutants for a subset of genes encoding the pathway and its maturation (Table S1) and compared their anaerobic growth to wild type (WT) when challenged with ferulic or *p*-coumaric acids (Fig. 3B). We also constructed and tested two CRISPRi partial knockdown strains of the *suf* operon using mismatched sgRNAs targeting *sufB*, the first biosynthetic gene downstream of *ZMO0422* (Fig. 3B; Fig. S9). Finally, to interrogate whether the mutations increased fitness to other phenolic acids, we also measured growth with vanillic acid. In untreated media, all deletion mutants grew similarly to WT, demonstrating that loss of these genes had no fitness cost during anaerobic growth in rich medium. As expected for essential gene perturbations, *suf* partial knockdown strains showed slight growth defects in untreated media compared to a non-targeting CRISPRi control strain. However, in the presence of any of the phenolic acids, including vanillic acid, all mutants grew more rapidly and/or to a higher terminal optical cell density (OD_600_) than their parent control (Fig. 3B). To further strengthen these results, complementation of the Δ*cytbc*_1_ and Δ*cytC* mutations with the corresponding WT alleles expressed from a plasmid reversed their fitness advantage in ferulic acid, returning to the fitness defect observed in WT (Fig. S10).

### Ferulic acid fails to induce a strong iron starvation response in *Z. mobilis*

**Fig 4:**
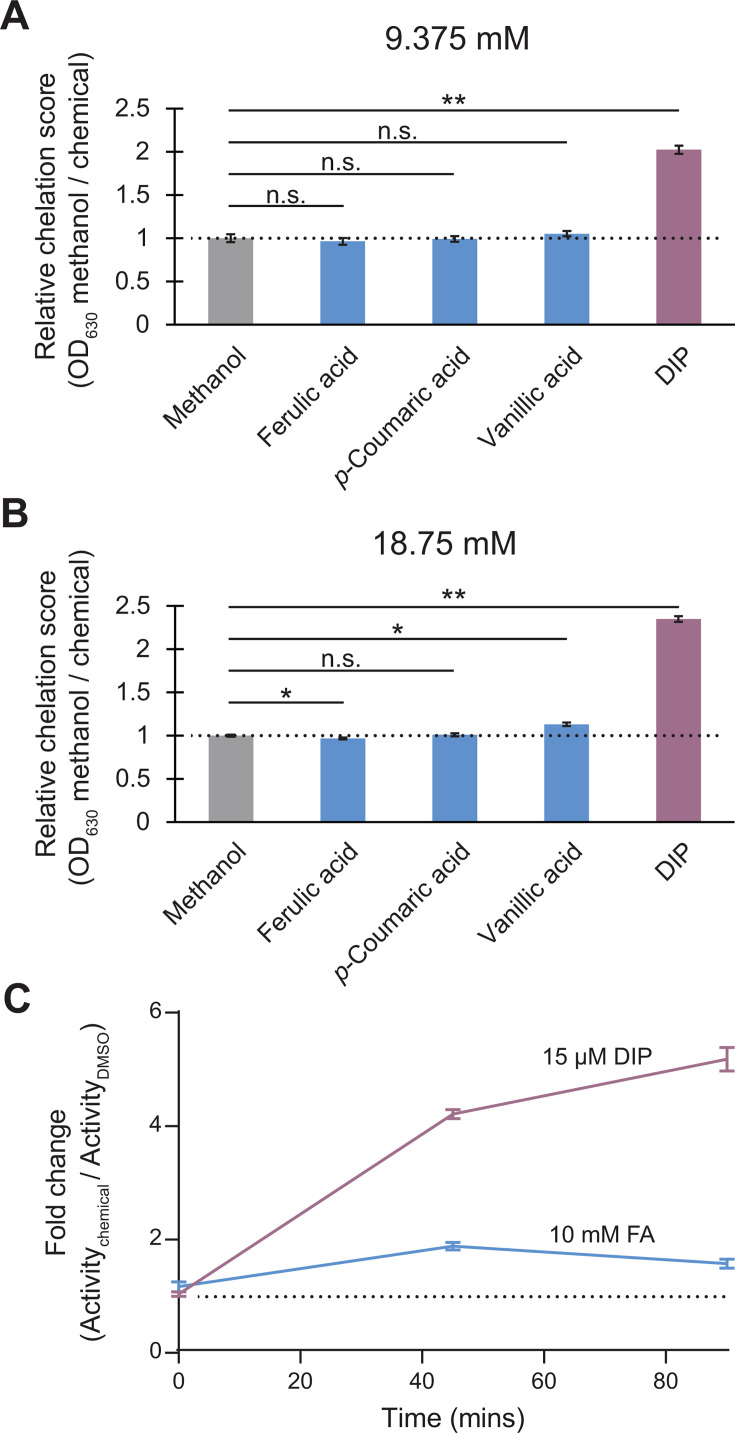
Phenolic acid iron chelation assays. (**A and B**) Results of an *in vitro* colorimetric CAS iron binding assay with chemicals at (**A**) 9.375 mM or (**B**) 18.75 mM, concentrations approximating those used in the chemical genomics screen and growth experiments (blue). The known metal chelator 2,2-dipyridyl (DIP) was used as a positive control (purple). Methanol was used as a solvent control (gray). The dashed line indicates the average of the solvent control. *, *P* ≤ 0.05; **, *P* ≤ 0.01; n.s., not significant (*P* > 0.05). (**C**) β-Galactosidase assay measuring *fhuE* promoter activity with ferulic acid or DIP. Activity is shown as fold change in Miller units between a 1% DMSO negative control and treatment. Error bars show the standard error of triplicate samples.

There is mixed evidence on whether phenolic acids can chelate iron, which could reduce iron bioavailability and perturb iron homeostasis (60, 61). Given that our target genes were iron-containing cellular components, we hypothesized that the improved fitness of our deletion and knockdown mutants in phenolic acids could result from sparing iron for other essential processes. To test the ability of ferulic, *p*-coumaric, and vanillic acids to chelate iron, we performed a chrome azurol S (CAS) iron binding assay (62). This assay showed negligible iron binding at concentrations approximating those used in our chemical genomics screen and mutant growth experiments (9.375–18.75 mM), except for a slight iron binding signal for 18.75 mM vanillic acid (Fig. 4A and B; Fig. S11). To examine possible iron stress directly in *Z. mobilis*, we measured expression of a *lacZ* transcriptional fusion to the iron-starvation responsive *fhuE (ZMO0188*) promoter (63); however, we observed only a minor increase in promoter activity with 10 mM ferulic acid, a concentration sufficient to slow growth and ~670× higher than concentration of the known iron chelator, 2,2′-dipyridyl, which efficiently induced *fhuE* expression (Fig. 4C). Overall, our results suggest that phenolic acids have a very minor effect on iron bioavailability.

### Ferulic acid causes proteome remodeling in the cell envelope

**Fig 5:**
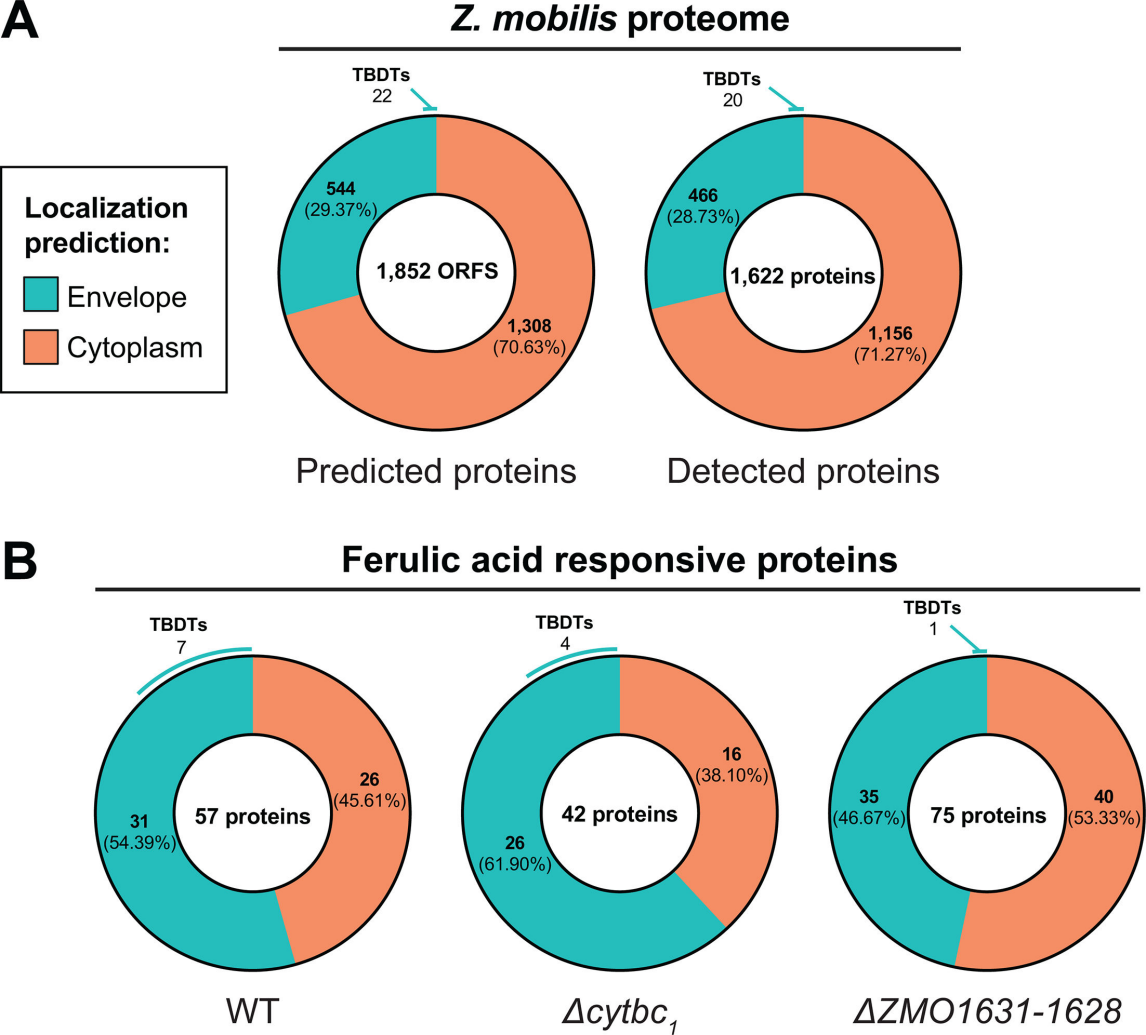
Proteome profiling of ferulic acid-treated *Z. mobilis*. (**A**) Protein localization prediction for the putative *Z. mobilis* proteome (left) and for proteins that were detected in at least one sample in the proteomics screen (right). (**B**) Protein localization prediction for proteins that had significant changes in abundance (FDR < 0.05, |LFC| > 1) in response to ferulic acid treatment in WT, Δ*cytbc_1_*, and Δ*ZMO1631-ZMO1628*.

To further investigate the cellular effects of phenolic acid-induced stress, we performed untargeted proteomics and compared protein abundance between untreated and ferulic acid-treated WT, Δ*cytbc_1_* (Δ*ZMO0956-ZMO0958*), and Δ*ZMO1631-ZMO1628*, the operon encoding proteins of unknown function. The three strains were grown anaerobically and treated with a sub-lethal concentration of ferulic acid. Samples for proteomics were collected just prior to treatment and 2 hours afterward. In total, our proteomic analysis detected peptides corresponding to the vast majority of predicted ORFs in *Z. mobilis* (1,622 proteins detected of the 1,852 predicted ORFs; Fig. 5A). Of the 230 proteins that were not detected, 134 were in the lowest 20% of anaerobically expressed proteins based on previously published transcriptomics data (64), leaving the possibility that they were too low in abundance to be detected (Table S6). Notably, only three of the 40 genes that had a significant phenotype with ferulic acid in our chemical genomics screen (Table S3) encoded proteins that were not detected in our proteomics assay. This includes the phenotypically relevant proteins ZMO1629 and ZMO1630, encoded by the *ZMO1631-ZMO1628* operon, which were not detected in any WT or Δ*cytbc_1_* samples. However, both ZMO1631 and ZMO1628 were detected, so these served as a proxy for the other operon members following ferulic acid addition. We compared protein relative abundance between samples treated with ferulic acid/DMSO or DMSO after 2 hours, revealing 57 proteins that had significantly changed abundance in response to ferulic acid in WT *Z. mobilis* (FDR < 0.05, |log_2_ fold change| > 1). The Δ*cytbc_1_* and Δ*ZMO1631-ZMO1628* mutants showed 42 and 75 significantly changed proteins, respectively (Fig. 5B). Of the proteins which had a significant change in abundance in at least one strain, only six had significant phenotypes in the chemical genomics screen in both Tn and CRISPRi, indicating that many of our proposed engineering targets to improve ferulic acid resistance are not responsive to ferulic acid under the tested conditions.

The overall proteomic response to ferulic acid was similar across the three strains tested, with many ferulic acid-responsive proteins predicted to be localized to the cell envelope (inner membrane, periplasm, outer membrane). We considered proteins containing a secretion signal sequence, e.g., Sec or Tat peptides that target proteins for secretion to the envelope (65), or a transmembrane domain consisting of either α-helical or β-barrel domains as envelope proteins and all other proteins as cytoplasmic (see Materials and Methods). Our analysis found that 544 of the predicted 1,852 ORFs (29.37%) in *Z. mobilis* encode proteins predicted to localize to the cell envelope (Fig. 5A; Table S4). Of the 1,622 proteins that were detected in all proteomics samples, 466 (28.73%) are predicted envelope proteins. Notably, envelope proteins made up a disproportionately large fraction of ferulic acid responsive proteins across all three strains (54.39% in WT, 61.90% in Δ*cytbc_1_*, and 54.39% in Δ*ZMO1631-ZMO1628*) (Fig. 5B). These percentages are significantly higher than would be expected by random chance (*P* = 0.0074, 0.0041, 0.0431 for WT, Δ*cytbc_1_*, and Δ*ZMO1631-ZMO1628*, respectively, Fischer’s exact test; Fig. S12), suggesting that ferulic acid stress targets the cell envelope.

### Transporter abundance is modulated in response to treatment with ferulic acid

**Fig 6:**
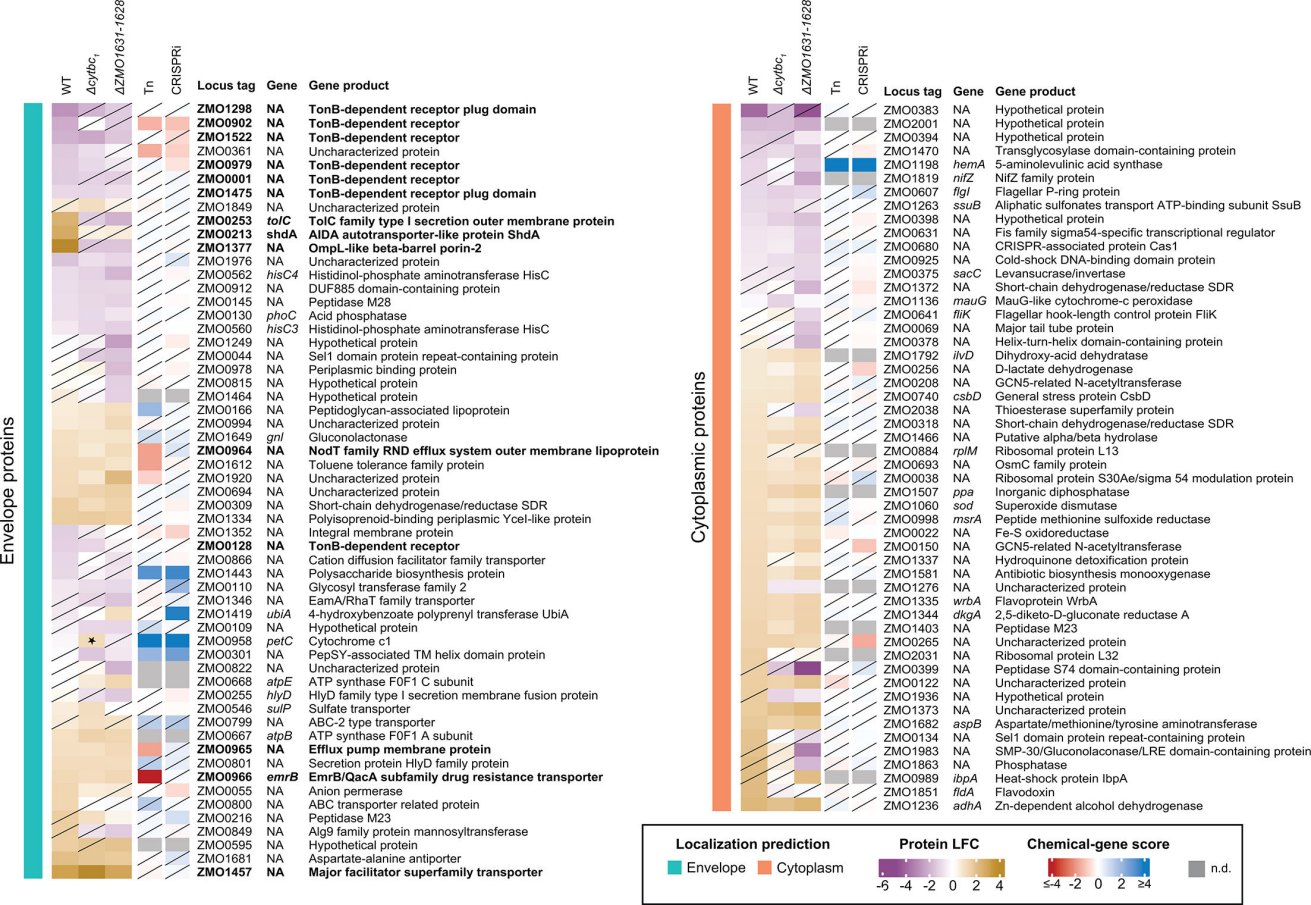
Heatmap of ferulic acid-responsive proteins. Heatmap of all genes that had significantly (FDR < 0.05, |LFC| > 1) changed abundance in at least one strain in response to ferulic acid treatment. Chemical-gene scores in 7.5 mM ferulic acid are displayed in the two right-most columns. Transporter proteins that are discussed in the main text are bolded. n.d., no data. Diagonal lines indicate statistical non-significance (FDR > 0.05). The starred protein is deleted in the associated strain and has a significant LFC due to missing value imputation during proteomic analysis.

To further identify trends in proteome remodeling in response to ferulic acid, we performed functional enrichment analysis (see Materials and Methods) (66) (Fig. S13, Table S5). We found that enrichments were similar across all three strains, indicating that the mutants respond to ferulic acid similarly to WT despite their increased phenolic acid resistance. These common enrichments include cell envelope proteins, oxidoreductase activity, and carbon metabolism. In particular, TonB-dependent transporters (TBDTs) were highly represented among the functional enrichments, suggesting a key role in responding to ferulic acid stress. TBDTs are proton motive force-coupled complexes that facilitate transport of large compounds, such as siderophores, vitamin B12, and glycans, across the outer membrane (67–69). Of the 22 annotated TBDTs in *Z. mobilis*, seven had significantly decreased abundance in at least one strain in response to ferulic acid (|log_2_ fold change| > 1, FDR < 0.05; Fig. 6).

Our proteomics data also revealed several transporters that increased abundance across all three strains in response to ferulic acid, including a predicted major facilitator superfamily transporter (ZMO1457) and a predicted efflux system (ZMO0964-ZMO0966) (Fig. 6; Table S6). Other transporters were responsive only in a single strain. For example, ZMO0253, ZMO0213, and ZMO1377 are transport-related proteins that increase abundance in WT in response to ferulic acid but were not responsive to ferulic acid in Δ*cytbc_1_* or Δ*ZMO1631-ZMO1628*. While transporters that increase in abundance in response to ferulic acid might be candidates for proteins involved in efflux of the chemical, none of the genes encoding these transporters showed consistently strong CG scores across the Tn and CRISPRi genetic screens (Fig. 6). This could be explained by possible functional redundancy among multiple transporters, though further study is required to genetically dissect this.

## DISCUSSION

Industrial microorganisms resilient to plant- and process-derived chemical stressors are required for efficient bioproduction. However, an incomplete understanding of the genetic basis for stress tolerance and susceptibility hinders rational engineering of robust strains. To identify genetic engineering targets, we developed an orthogonal and parallel screening strategy using Tn and CRISPRi libraries that leverages the complementary strengths of both technologies to identify target genes with high specificity. By applying our strategy to investigate the effects of inhibitory chemicals on *Z. mobilis* during anaerobic growth, we narrowed the genetic search space from 103 genes identified by at least one library to 31 cross-validated engineering targets. All follow-up mutants displayed the predicted chemical resilience phenotypes, indicating that our strategy precisely identifies true positives. Our combined approach is readily applicable to a wide range of bacteria with implications across industrial, medical, and environmental research—especially where reducing frequency of false hits is highly desirable—and could be immediately implemented for species with existing CRISPRi and Tn libraries, such as *E. coli, Bacillus subtilis,* or *Streptococcus pneumoniae* (49, 70–74).

This work builds on prior investigations by characterizing chemically mediated stress in production-relevant anaerobic conditions. Previously, a transposon screen conducted aerobically identified gene disruptions that decrease fitness in common feedstock inhibitors, including ferulic and *p*-coumaric acids (15). Among these are genes encoding cytochrome *c* maturation (*ccmF, ZMO1255; ccmI, ZMO1252; ccmH, ZMO1253*) and Fe–S cluster biosynthesis (*sufE, ZMO1067; sufA, ZMO0429*). In contrast, we found that disrupting these genes improved fitness with ferulic and *p*-coumaric acids under anaerobic conditions. Given that *Z. mobilis* displays distinct physiology depending on oxygen and nutrient availability (15, 59, 75), this contrast is likely due to differences in growth conditions (e.g., aerobic versus anaerobic growth and media composition). Our identification of more conditionally beneficial gene disruptions than Skerker and Leon et al. may also reflect our use of contemporary quantification technologies (next-generation sequencing instead of microarrays) which improved our ability to measure mutant abundance.

Our dual-library approach revealed that disrupting the cyt *bc*_1_/cyt *c* electron transport pathway improves fitness of *Z. mobilis* in phenolic acids under anaerobic conditions, indicating an unexpected anaerobic function for this pathway. In mitochondria and many bacteria, this pathway is well characterized for its role in aerobic respiration, where it transfers electrons to cytochrome *c* oxidase. However, *Z. mobilis* lacks a cytochrome *c* oxidase and instead uses a ubiquinol-cytochrome *bd* oxidase to reduce O_2_. Despite this, it has been shown that disruption of the cytochrome *bc*_1_ complex results in a decrease in O_2_ consumption, making the aerobic function of this pathway unclear (76, 77). An anaerobic function for the cyt *bc*_1_/cyt *c* pathway has been established in a few bacteria that carry out anaerobic respiration. For example, in *Pseudomonas aeruginosa,* electrons can be transferred from the cyt *bc*_1_/cyt *c* pathway to nitrite, nitric oxide, or nitrous oxide via associated reductases during anaerobic growth (78). However, no equivalent reductases are annotated in *Z. mobilis*, nor did our screen reveal any terminal reductase candidates, leaving the anaerobic function of this pathway an area for further investigation.

Our study also identified an operon of unknown function (*ZMO1631-ZMO1628*) with anaerobic phenotypes similar to cyt *bc*_1_/cyt *c* pathway phenotypes, suggesting a functional relationship. *ZMO1629* and *ZMO1630* had significant chemical-gene scores in both Tn and Ci libraries, and deletion of the entire operon confers increased fitness to ferulic and *p*-coumaric acids. The operon is predicted to be regulated by the iron starvation-responsive transcription factor, Fur (79), suggesting that it could play a role in iron acquisition and retention. Indeed, *ZMO1631* is a predicted TonB-dependent receptor, whose function is often associated with siderophore-mediated iron transport. AlphaFold3 modeling predicts that *ZMO1630-1628* forms a 12-transmembrane helix complex, with *ZMO1628* also containing a predicted PepSY domain, suggesting a possible function in transport. Moreover, other iron-regulated PepSY-domain containing proteins from *P. aeruginosa* and *Bradyrhizobium japonicum* have been identified as cytoplasmic membrane-embedded ferric reductases, which are involved in siderophore-mediated iron uptake (80–82). Thus, it is possible that this gene cluster may have a similar function in iron acquisition in *Z. mobilis*.

We further characterized the phenolic acid stress response using proteomics and found that ferulic acid causes remodeling of the cell envelope proteome, which is consistent with previous literature reporting that phenolic acids can perturb cell membranes (5, 83, 84). Furthermore, ferulic acid treatment results in changes in the abundance of several transporters, including putative efflux systems and TonB-dependent transporters, suggesting a potential role in ferulic acid transport. In the closely related genus *Sphingobium*, TonB was shown to play a role in the uptake of ferulic acid and other lignin-derived phenolics for subsequent catabolism (85, 86). However, specific TBDRs involved in ferulic acid uptake were not identified. *Z. mobilis* is not known to catabolize phenolic acids, and while *tonB* (*ZMO1717*) was essential in our Tn library—prohibiting further phenotypic analysis in our comparative screen—CRISPRi library partial knockdowns of *tonB* with mismatched sgRNAs had statistically insignificant ferulic acid phenotypes (Table S2). Consequently, it is possible that if TBDTs are involved in phenolic acid uptake in *Z. mobilis*, it is as an unintended substrate. Interestingly, the Δ*ZMO1631-ZMO1628* mutant, which harbors a deletion for the annotated TBDT gene *ZMO1631*, had fewer significantly downregulated TBDTs than the WT or Δ*cytbc_1_* strains. It is possible that this deletion might also provide resistance by decreasing chemical import. Although our genetic screens did not pinpoint other TBDTs with phenolic acid resistance phenotypes, this could be explained by redundant functions among multiple TBDTs. Finally, recent work in *Z. mobilis* also found that TBDTs displayed decreased abundance when grown in Ammonia Fiber Expansion pretreated biomass (87), indicating that this regulatory response occurs not only during growth with purified ferulic acid but also in complex feedstocks, where TBDTs may be involved in transporting a variety of compounds.

Our orthogonal screening approach robustly identified genes that provided accurate engineering targets. However, genes that fell below our cutoff and were identified only by CRISPRi or Tn insertion may also be beneficial engineering targets. Thus, if a more comprehensive view of all potential targets is desired, then adjusting the cutoffs to be more permissive is straightforward but will need to be counterbalanced by screening more false positives and utilizing more resources. CRISPRi and Tn libraries are known to have certain limitations in associating phenotypes with particular genes, making the dual approach used here so effective in minimizing false positives. In the case of CRISPRi, off-target sgRNA binding or toxic sgRNA sequences (88) can produce incorrect phenotypes. For Tn insertions, multiple insertions per genome (89) or stabilization of merodiploid/polyploid states of the chromosome caused by insertions in essential genes (15, 90, 91) can also obscure phenotype assignments. Finally, differing *cis* effects on downstream gene expression between the two techniques may also yield different gene phenotypes, revealing interesting biology. For example, while Tn insertion in *ZMO0422*—the first gene in the *suf* operon and the only one not directly performing iron–sulfur cluster assembly—had an insignificant phenotype with phenolic acids, *ZMO0422* CRISPRi knockdowns were conditionally beneficial. Given that our Mismatch-CRISPRi partial knockdowns of essential *suf* genes also provided a conditional relative fitness advantage, the CRISPRi phenotype for *ZMO0422* is likely due to CRISPRi polarity onto downstream *suf* genes.

In conclusion, we developed an approach for rapid identification of high-value gene targets and used it to advance our understanding of *Z. mobilis* chemical tolerance in anaerobic conditions, providing valuable insights for future bioengineering efforts. We highlight the cyt *bc*_1_/cyt *c* pathway as a promising target for engineering robust biofuel production strains with improved phenolic acid resistance, particularly in plant-based feedstocks with high concentrations of phenolics (4, 92, 93). Looking ahead, we anticipate that adaptations of our comparative approach will accelerate discoveries in fields driven by chemical-gene interactions, including industrial biomanufacturing, combating antibiotic resistance, and understanding chemically-mediated symbioses within human or plant microbiomes.

## MATERIALS AND METHODS

### Bacterial growth conditions

*Z. mobilis* was grown at 30°C in either *Zymomonas* rich defined medium (ZRDM) or rich medium glucose (ZRMG) (59). Anaerobic growth was achieved either by culturing in a Coy chamber with 10% CO_2_, 5% H_2_, and a balance of N_2_ or by sparging with 95% N_2_ and 5% CO_2_. *E. coli* was grown in Lennox broth at 37°C. Where indicated, a final concentration of 100 µg/mL ampicillin (amp), 20 µg/mL chloramphenicol (cm), or 100 µg/mL spectinomycin (spec) was added to *E. coli* media, and 120 µg/mL cm or 100 µg/mL spec to *Z. mobilis* media. A final concentration of 300 µM diaminopimelic acid was added for the growth of Dap^−^ *E. coli* mating strains. Where indicated, CRISPRi libraries and strains were grown with 1 mM isopropyl β-D-1-thiogalactopyranoside (IPTG); complementation strains were grown with 50 µM IPTG.

For anaerobic growth measurements in a Coy chamber, 24-well plates containing 1.2 mL ZRDM supplemented with 1% (vol/vol) DMSO or phenolic acid dissolved in DMSO (100×) were inoculated after equilibration. Strains were grown to saturation, and plates were inoculated with saturated cultures to a final OD_600_ of 0.05. Growth of strains was analyzed in quadruplicate for each condition by measuring OD_600_ of each well every 30 minutes for 72 hours using a Tecan Spark plate reader equipped with a stacker.

### Construction of bacterial strains and libraries

Strains, plasmids, and oligonucleotides are listed in Table S1. Deletion mutants were constructed via homologous recombination, and recombinants were identified by *gfp* screening as described previously (31) or using *sacB* counterselection. Briefly, for *gfp* screening, a non-replicative plasmid (PK15534) encoding *cat* (chloramphenicol resistance), *gfp*, and ~500 bp flanking regions from upstream and downstream of the gene targeted for deletion was conjugated from *E. coli* WM6026 into WT *Z. mobilis*, where it recombined into the genome. Conjugants are selected by plating on ZRMG and chloramphenicol. Subsequent recombination of the plasmid out of the genome was identified by loss of GFP fluorescence via fluorescence-activated cell sorting, and final recombinants were screened by colony PCR to distinguish mutants from WT.

For *sacB* counterselection, *gfp* of PK15534 was replaced with *sacB*, encoding levansucrase from *B. subtilis*, which confers sucrose sensitivity in Gram-negative bacteria (94). The PK15534 backbone was PCR amplified with homologous overhangs using primers “PK15534 bb noGFP fwd/rev,” and *sacB* was amplified from pJQ200SK (95) using primers “*sacB* fwd/rev” (Table S1). Gibson assembly yielded pPK17311, which was then engineered with homologous flanking regions and conjugated into WT *Z. mobilis*. Conjugants were selected by plating on ZRMG and chloramphenicol before being grown overnight without antibiotics and then plated on ZRMG 5% sucrose plates to counterselect for recombination of the plasmid out of the genome. To confirm successful plasmid excision, colonies were streaked on ZRMG plates with and without chloramphenicol (120 µg/mL), and chloramphenicol-sensitive colonies were screened via colony PCR to differentiate mutants from WT. All deletion mutants were verified by whole-genome resequencing using NextSeq1000 (96).

Genetic complementation of Δ*cytbc_1_* and Δ*cytC* mutants (PK16845 and PK16846) was performed using pPK16904, a pRL814-derived expression vector (97) carrying the IPTG-inducible promoter B from pJMP2093 (33). The plasmid backbone was amplified using “pPK16904_backbone_fwd/rev” primers, and the coding sequences for *ZMO0956-ZMO0958* (*cytbc_1_*) and *ZMO0961* (*cytC*) were amplified from the *Z. mobilis* genome using “cytbc1_fwd/rev” and “ZMO0961_fwd/rev,” respectively (Table S1). After Gibson assembly, plasmids were transformed into *E. coli* WM6026 and conjugated into their complementary deletion strains, resulting in PK16854 (Δ*cytC* pPK16849) and PK16855 (Δ*cytbc_1_* pPK16852).

Individual Mobile-CRISPRi mutants were constructed as described previously (33, 98). Briefly, sgRNA-encoding sequences were cloned between BsaI sites of the Mobile-CRISPRi vector (pJMP2656), and the Mobile-CRISPRi system was transferred to the *att*Tn*7* site of the *Z. mobilis* chromosome via conjugation using *E. coli* WM6026-derived strains from Ward et al. (Table S1) (99).

The *Z. mobilis* Tn*5* insertion library was constructed in ZRMG under aerobic conditions as previously described (100). The *Z. mobilis* Mobile-CRISPRi library was constructed previously and consists of three sub-libraries: (i) sJMP2618, Z1-genes, perfect-match spacers; (ii) sJMP2619, Z2-controls, non-targeting spacers; and (iii) sJMP2620, Z3-mismatches, mismatched spacers (59).

### Chemical genomics screen

Chemical genomics screens were performed anaerobically, and trace O_2_ was removed by placing all components inside the Coy anaerobic chamber for at least 18 hours before inoculation. Relevant concentrations of chemicals and suppliers are listed in Table S1. Feruloyl amide and coumaroyl amide were synthesized as previously described (4). Chemical concentrations that caused ~10%–40% reduction in the empirical area under the growth curve, as calculated by the Growthcurver R package (v0.3.1), for *Z. mobilis* sJMP2554 (non-targeting CRISPRi strain) compared to the untreated control were used in the screen (101).

The *Z. mobilis* CRISPRi library (sJMP2618, 2619, 2620) inoculum was prepared as follows: sub-libraries Z1, Z2, and Z3 were mixed at a ratio of 8:1:6, and 400 µL total library was added to 40 mL ZRDM and grown at 30°C with stirring to OD_600_ ~0.5. Untreated control samples were collected for sequencing, and cells from this flask were used to inoculate 1.5 mL ZRDM with 1 mM IPTG in the presence or absence of chemical to an OD_600_ ~0.01 in 24-well plates and grown in a Tecan Spark multimode microplate reader with Spark Stacker, shaking for 10 seconds every 30 minutes, to the stationary phase (~46 hours). To allow sufficient generations for depletion of gene product following induction of the CRISPRi system with IPTG, the process was repeated by sub-culturing into a second 24-well plate and growing from a starting OD_600_ ~0.01 for another ~46 hours. At this time, CRISPRi library samples with ~8%–30% reduction in the empirical area under the growth curve (101) compared to the untreated controls were harvested for sequencing by centrifuging and storing cell pellets at −20°C.

The *Z. mobilis* Tn5 transposon library (PK15455) was screened similarly, except that no IPTG was added to growth media, and 100 µL of the library was initially diluted in 10 mL ZRDM to an OD_600_ ~1.8 before transferring 75 µL to each 24-well plate for a starting OD_600_ ~0.09. Growth and subculturing into the second 24-well plate was performed as was done for the CRISPRi library. Samples were collected for sequencing at the same time points as CRISPRi samples by centrifuging and storing cell pellets at −20°C.

### Tn library sequencing and data analysis

Tn library genomic DNA was extracted using Qiagen DNeasy Blood and Tissue Kit (Qiagen, 69504), fragmented using a Covaris S220 Focused-Ultrasonicator, and converted to blunt-end DNA using NEBNext End Repair Module (NEB, E6050S) (100). C-tailing was performed on blunt-end DNA fragments using dCTP/ddCTP and Terminal Deoxynucleotidyl Transferase according to manufacturer guidance (Promega, M1871). Custom Tn RT fwd and Tn RT rev primers (Table S1) were used to PCR amplify C-tailed fragments to facilitate subsequent attachment of IDT for Illumina DNA/RNA UD Indexes (Illumina, Ref: 20026121, Lot: 20647691). Libraries were sequenced using NextSeq1000 (2 × 150 bp) (Illumina, 20046813). Two biological replicates were sequenced per condition.

Tn insertion sequencing data were pre-processed to remove transposon sequences using cutadapt (v3.4) (102) with default parameters from the R1 FASTQ file. After trimming, the sequencing data were aligned to the *Z. mobilis* ZM4 genome (GCF_003054575.1-RS_2023_03_19) using bowtie (v1.3.1) (103) and default parameters. Transposon insertion sites were identified with TSAS (v2.0) (104) using the one-sample analysis mode with minimum hits set to 5, clipping set to 5, capping set to 0, and weights set to 0. Essential genes in the absence of chemical treatment were classified by *P* <0.05 and normalized unique hits per bp <0.025 in the untreated and solvent control conditions (Table S7). Changes in fitness (i.e., chemical-gene scores) were identified using pairwise comparison in edgeR (v4.2.0) (105) to compare the number of insertions per gene in the experiment to the control samples.

### CRISPRi library sequencing and data analysis

Two biological replicate CRISPRi samples were sequenced per condition, with the exception of the cinnamic acid conditions, which had one replicate per concentration. DNA was extracted from cell pellets using the Thermo Scientific GeneJET Genomic DNA Purification Kit (K0721), eluting in a final volume of 50 µL with typical yields of ~10–50 ng/µL. The sgRNA-encoding region was amplified by low-cycle Q5 PCR (19 cycles) as described previously (59), using primers oJMP697/oJMP698 or barcoded primers oJMP1678-1773/oJMP698 (106). PCR products were spin purified, and samples were sequenced by the UW-Madison Biotechnology Center Next Generation Sequencing Core facility using a NovaSeq 6000 (150 bp paired-end reads), at least 20 million reads per sample, and 20%–30% PhiX when necessary for sequence diversity.

Pooled sample sequencing data were demultiplexed by primer barcode using cutadapt (v4.2) (102). As performed previously (59), sgRNA-encoding spacer sequences were counted using the seal.sh script (v38.90) from the BBTools package (https://sourceforge.net/projects/bbmap/), and counts were compared using edgeR (v.4.0.16) to calculate chemical-gene scores (log_2_ fold changes between treated and untreated conditions, LFC) and corresponding false discovery rates (105). Gene-level scores were calculated as the median LFC of perfect-match spacers targeting each gene, and gene-level significance was determined using Stouffer’s *P* value (poolr R package v1.1-1) based on the FDRs of the spacers (107).

Essential genes required for viability in the absence of chemical treatment were classified by gene-level significance ≤0.05 and median LFC ≤−5.1 in the IPTG-induced final time point (T2_induced_) compared to the uninduced initial time point (T0_uninduced_) (Table S7). This median LFC cutoff was chosen to approximate a similar fitness cost to that used to define *Z. mobilis* essential genes previously (59). Since median LFCs in this study were generally stronger than those reported previously, likely due to longer outgrowth following induction, a linear regression (*y* = 1.567*x* − 0.373) was calculated representing the relationship between gene LFCs in each study. This regression was used to extrapolate the essential gene cutoff from Enright et al. (59) (LFC ≤ −3) to the cutoff used in this work (LFC ≤ −5.1).

### Comparative Tn and CRISPRi analyses and visualization

Quantile normalization of Tn and CRISPRi chemical-gene scores was performed within each condition for all non-essential genes using the preprocessCore R package (version 1.64.0) (108). The pROC R package (version 1.18.5) was used to create ROC curves and calculate AUC (108). Heatmaps were generated using the ComplexHeatmap R package (version 2.18.0) (109). Hierarchical clustering was performed using Ward’s method. For essential *suf* genes, mismatched sgRNAs causing a partial fitness defect in the absence of chemical treatment, characterized by −4 ≤ LFC(T2_induced_/T0_uninduced_) ≤ −1 and FDR ≤ 0.05, were considered. The median chemical-gene score for these partial knockdown sgRNAs was calculated for visualization in heatmaps.

### Chrome azurol S liquid assay for iron-binding compounds

The CAS assay protocol was modified from reference 110 (version 2). Chemicals dissolved in methanol were mixed 1:1 with CAS solution in a clear 96-well microplate and incubated in the dark for 1 hour at room temperature. Absorbance at 630 nm was measured using a Tecan Infinite M Nano plate reader. The relative chelation score was calculated as the OD_630_ ratio of methanol to chemical.

### β-Galactosidase assay

The *fhuE::lacZ* promoter fusion strain (PK17304) was grown at 30°C in ZRDM anaerobically via sparging (95% N_2_ and 5% CO_2_). At OD_600_ 0.25, 2,2′-dipyridyl (DIP) or ferulic acid was added to a final concentration of 15 µM and 10 mM, respectively. DMSO was included as a solvent control. Cell samples were collected for enzymatic assay prior to and 45 and 90 minutes after chemical treatment by adding chloramphenicol to a final concentration of 120 µg/mL to halt cell growth and protein synthesis before being stored on ice. β-galactosidase activity was then assayed to measure *fhuE* promoter activity (111).

### Proteomics sample growth and preparation

Strains were grown anaerobically in ZRDM via sparging to approximately OD_600_ ~0.3 before being treated with 1:100 (vol/vol) DMSO or 1 M ferulic acid in DMSO (final concentration in culture 10 mM). Each treatment had three replicates for each strain. Proteomics samples were harvested prior to and 2 hours after treatment by collecting 1 mL culture, pelleting cells, and freezing the cell pellet with dry ice before storing at −80°C.

Proteins were precipitated from *Z. mobilis* cells with 2:2:1 methanol:acetonitrile:H_2_O, then centrifuged at 12,000 × *g* for 10 minutes. Protein pellets were then dissolved in a lysis buffer containing reducing and alkylating agents (8 M urea, 100 mM Tris-HCl, pH 8.0, 10 mM TCEP, 40 mM CAA). Protein concentration was quantified using BCA (Thermo) and brought to 1.5 mg/mL. Sequencing grade LysC (Wako) and trypsin (Promega) were added to the samples simultaneously, both at 50:1 protein:protease, and the samples were incubated overnight at room temperature. Samples were acidified with formic acid (FA) to pH 2, then desalted using Strata-X 33 µm Polymeric Reversed Phase cartridges. Peptides were then dried by speedvac and reconstituted in 0.2% FA, quantified by Nanodrop (A205), and brought to 1 mg/mL.

### Liquid chromatography mass spectrometry-based proteomics and data analysis

For each sample, 500 ng of peptide was injected onto a 75 µm ID fused silica column packed (112) with 1.7 µm, 130 Å pore size, Bridged Ethylene Hybrid-C18 particles (Waters) using a Vanquish Neo (Thermo Scientific). The column was maintained at 50°C inside an in-house-made heater. Peptides were analyzed across a 74 minute separation where the mobile phase was ramped from 100% mobile phase A (0.2% FA) to 46% mobile phase B (80% acetonitrile/0.2% FA/19.8% H_2_O) before washing with 100% mobile phase B and then equilibrating with 100% mobile phase A.

Mass spectra were acquired using an Orbitrap Ascend tribrid mass spectrometer (Thermo Scientific) with data-dependent acquisition (DDA). MS1 data were collected with the Orbitrap at a resolving power of 240,000 at 200 *m/z* from 300 to 1,350 *m/z*, and an AGC target of 250%. With a cycle time of 1 second, MS2 data were collected on peptides of charge state 2–5 in the linear ion trap (scan rate Turbo) using advanced precursor determination (113) over a scan range of 150–1,350 *m/z*, and 250% AGC with 12 ms max inject time. Monoisotopic precursor selection was used in peptide mode, and dynamic exclusion was applied to ions for 20 seconds with a mass tolerance of ±5 ppm. DDA proteomics data were analyzed with MSFragger (version: 22.0) using default settings and the *Z. mobilis* ZM4 reference genome (downloaded from Uniprot on 30 September 2024).

### Protein localization prediction

SignalP 6.0 was used to test the *Z. mobilis* proteome for proteins containing a Sec or Tat signal peptide indicative of cell envelope or extracellular localization (114). Proteins predicted to have a signal peptide with a likelihood greater than 0.9 were considered an envelope protein. To identify proteins that lack a signal sequence but have a transmembrane domain, the proteome was screened using DeepTMHMM 1.0 (115). Proteins that had either a signal peptide or a transmembrane domain were annotated as envelope proteins. All other proteins were categorized as cytoplasmic.

### STRING functional enrichment analysis

The STRING database (v12.0) (66) was used to identify functional patterns in our proteomics data. For each strain, protein names and their corresponding log_2_ fold change scores before and 2 hours after ferulic acid exposure were submitted to the Proteins with Values/Ranks feature on the STRING website (string-db.org). Local Network Clusters were used to identify patterns between strains.

## Data Availability

Raw sequencing data for Tn and CRISPRi samples are available through the NCBI BioProjects PRJNA1270032 and PRJNA1276497, respectively. Raw proteomics data are available in the MassIVE database (MSV000097906). Code used for this work is available at https://github.com/GLBRC/orthogonal-chemgen-approaches-in-zmo.

## SUPPLEMENTAL MATERIAL

Fig. S1 to S3 msystems.01001-25-s0001.pdf Fermentation-relevant chemicals, box plots, and ROC curves.

Fig. S4 msystems.01001-25-s0002.pdf Hierarchical clustered heatmap of all non-essential genes and their associated chemical-gene scores in every condition.

Supplemental figures msystems.01001-25-s0003.pdf Fig. S5 to S13.

Table S1 msystems.01001-25-s0004.xlsx Strains, oligonucleotides, plasmids, and chemicals.

Table S2 msystems.01001-25-s0005.xlsb All library fitness data.

Table S3 msystems.01001-25-s0006.xlsx Putative engineering targets.

Table S4 msystems.01001-25-s0007.xlsx Protein localization predictions.

Table S5 msystems.01001-25-s0008.xlsx All STRINGS clusters from proteomics.

Table S6 msystems.01001-25-s0009.xlsx Ferulic acid proteomics data.

Table S7 msystems.01001-25-s0010.xlsx Essential genes for anaerobic growth in ZRDM.

ASM does not own the copyrights to Supplemental Material that may be linked to, or accessed through, an article. The authors have granted ASM a non-exclusive, world-wide license to publish the Supplemental Material files. Please contact the corresponding author directly for reuse.
