# Supplementary material for: Orthogonal chemical genomics approaches reveal genomic targets for increasing anaerobic chemical tolerance in Zymomonas mobilis
Source: mSystems. 2025 Dec 4;11(1):e01001-25. doi: 10.1128/msystems.01001-25 (PMC12817903; doi:10.1128/msystems.01001-25)
Supplement: Fig. S1 to S3 — Fermentation-relevant chemicals, box plots, and ROC curves. [file msystems.01001-25-s0001.pdf]

Chemical name	Structure	Concentrations tested
Ferulic acid	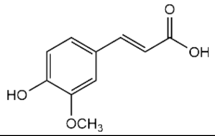	0.46875 mM, 7.5 mM, 11.25 mM
p-Coumaric acid	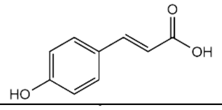	6.25 mM, 7.5 mM, 9.375 mM
Feruloyl amide	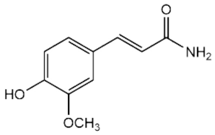	10 mM, 15 mM
Coumaroyl amide	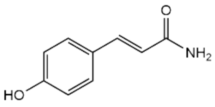	0.40625 mM, 13 mM, 15 mM
Cinnamic acid	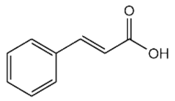	0.9375 mM, 1.875 mM, 3.75 mM
GVL	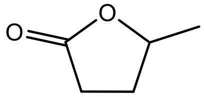	0.63%, 0.9375%
Furfural	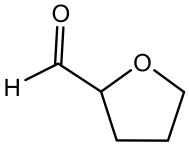	20 mM, 25 mM, 40 mM
Isobutanol	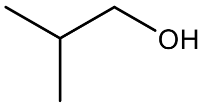	1.25%, 5%, 7.5%, 10%
Ethanol	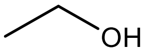	4%, 8%, 10%, 16%

Figure S1. Fermentation-relevant chemicals tested in orthogonal chemical genomics experiment.

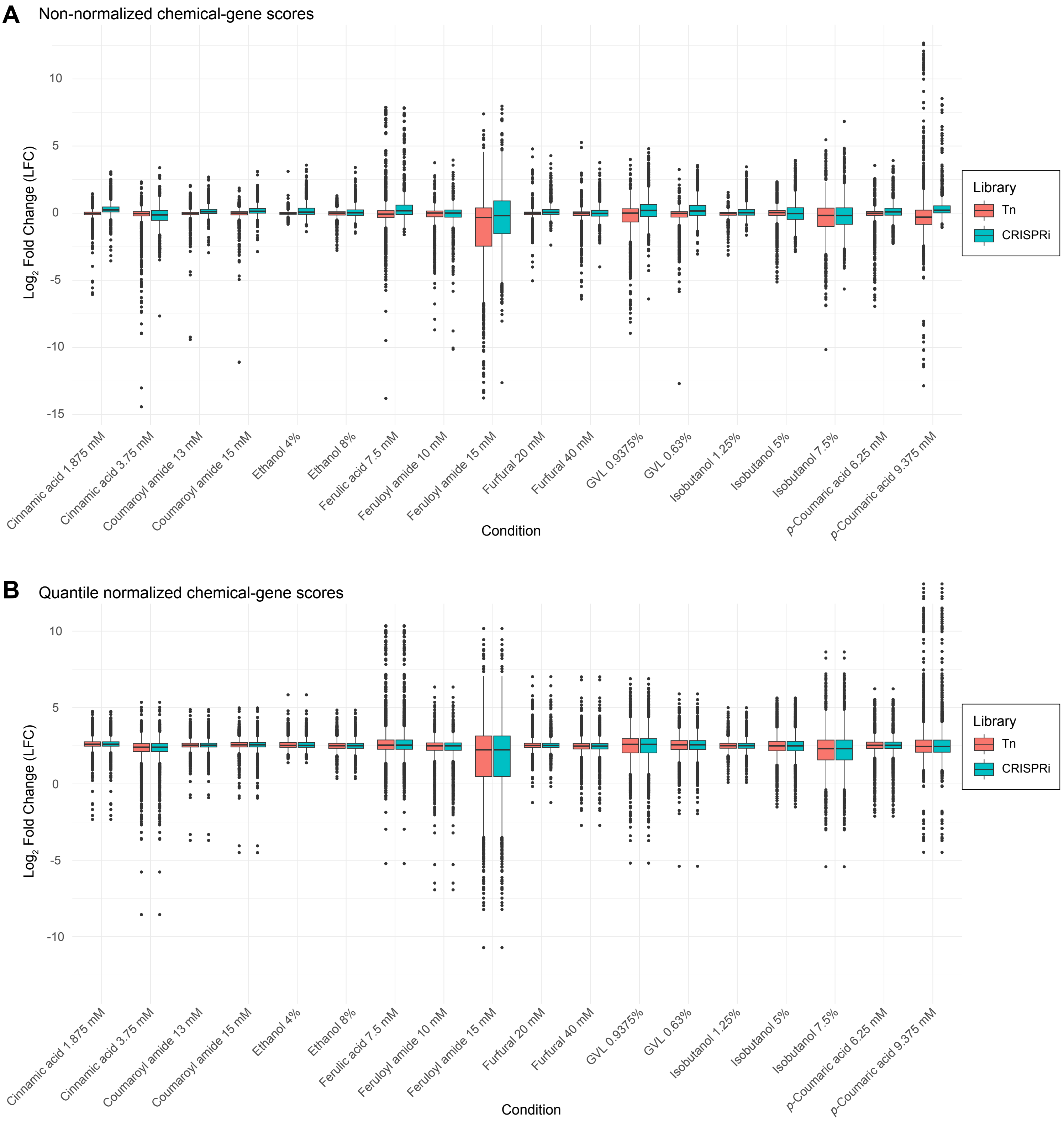

Figure S2. Box plots comparing the spread of chemical-gene scores between Tn and CRISPRi libraries in different chemical conditions prior to (A) and after (B) quantile normalization.

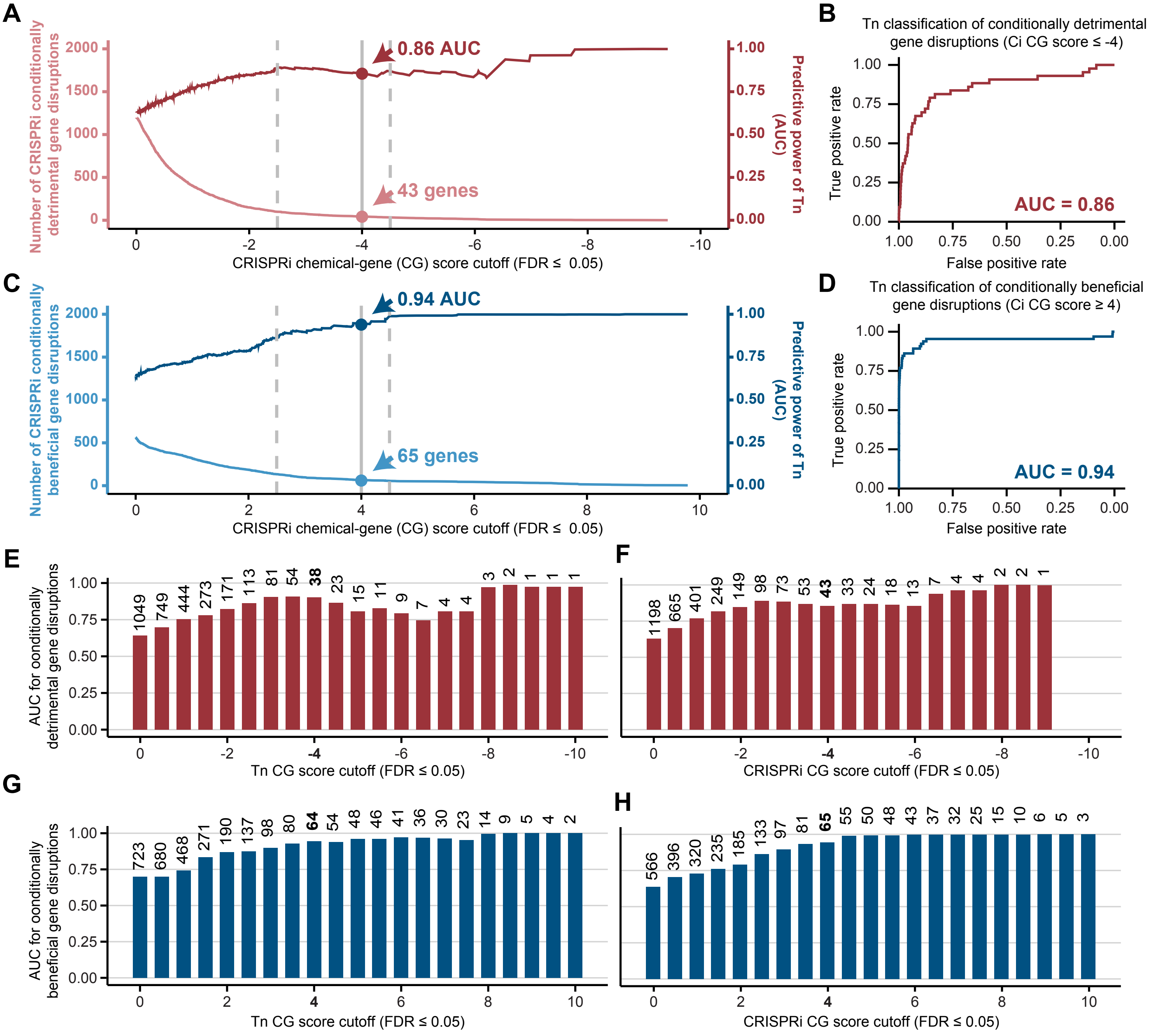

Figure S3. A and C) Comparing ROC curve area under the curve (AUC) at different chemical-gene (CG) score cutoff values for conditionally detrimental **(A)** or conditionally beneficial **(C)** gene disruptions when using the CRISPRi library as ground truth. The darker line depicts AUC at a given CG score cutoff (right y-axis) while the lighter line represents the number of genes which pass the associated cutoff value (left y-axis). Dashed vertical lines approximate the bounds of a flexible range within which researchers may choose to select cutoffs by weighing the tradeoff between number of hits and reliability of those hits. The solid gray line at |CG score| = 4 marks the score cutoff chosen in this work to identify genes for further study. **B and D)** ROC curves generated using **(B)** CG score ≤ -4 and **(D)** CG score ≥ 4 using the Tn library as ground truth. **E-H)** Bar plots summarizing the AUC at various whole integer CG score cutoffs. The number of genes passing each cutoff is listed on top of each bar. Plots are related to Fig 1B **(E)**, Fig S3A **(F)**, Fig 1D **(G)**, and Fig S3B **(H)**.
