# Supplementary figures and images for: Orthogonal chemical genomics approaches reveal genomic targets for increasing anaerobic chemical tolerance in Zymomonas mobilis
Source: mSystems. 2025 Dec 4;11(1):e01001-25. doi: 10.1128/msystems.01001-25 (PMC12817903; doi:10.1128/msystems.01001-25)

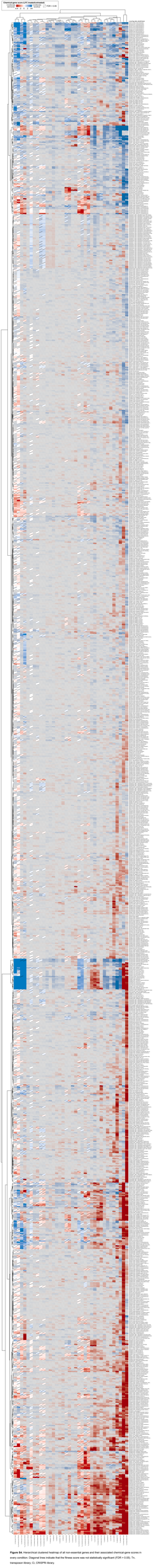

Supplement: Fig. S4 — Hierarchical clustered heatmap of all non-essential genes and their associated chemical-gene scores in every condition. [file msystems.01001-25-s0002.pdf]
