# Supplementary material for: Orthogonal chemical genomics approaches reveal genomic targets for increasing anaerobic chemical tolerance in Zymomonas mobilis
Source: mSystems. 2025 Dec 4;11(1):e01001-25. doi: 10.1128/msystems.01001-25 (PMC12817903; doi:10.1128/msystems.01001-25)
Supplement: Supplemental figures — Fig. S5 to S13. [file msystems.01001-25-s0003.pdf]

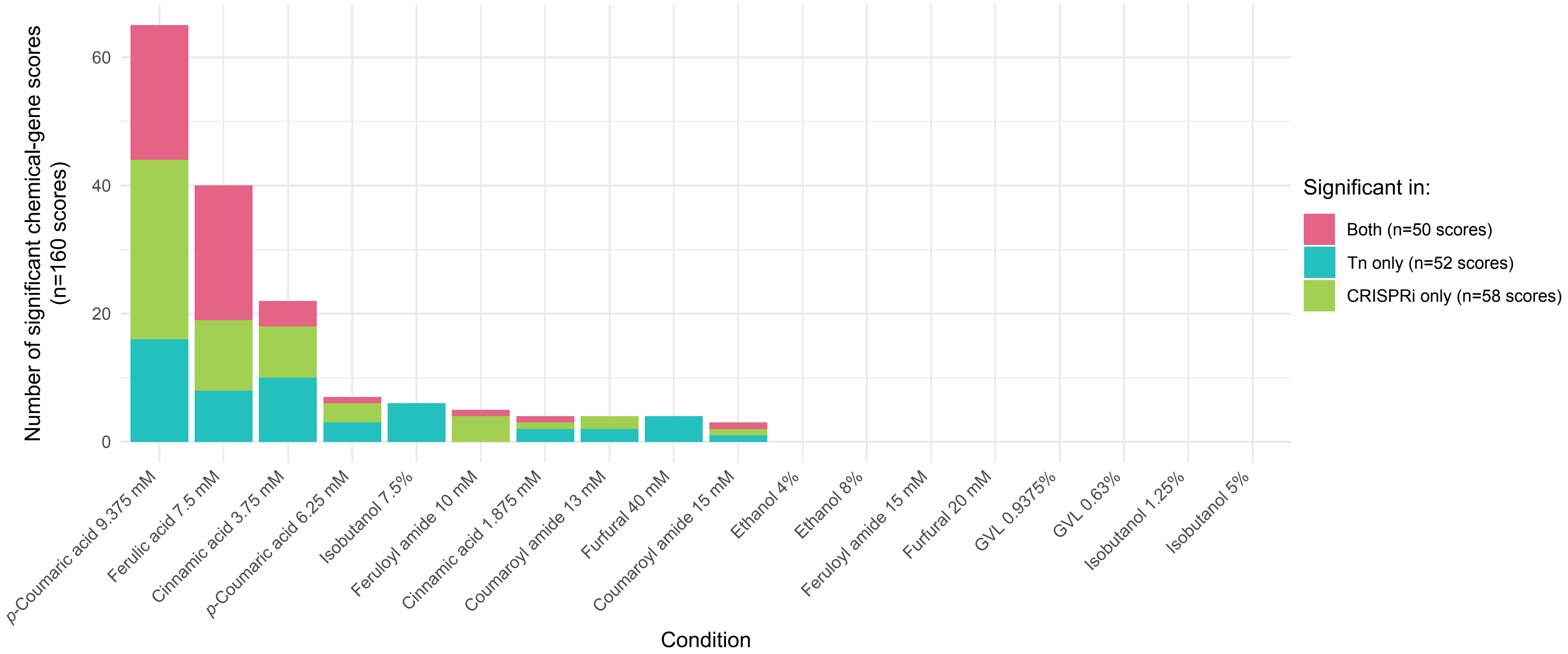

Figure S5. Bar plot depicting the number of significant hits (|LFC| ≥ 4 and FDR ≤ 0.05) in either or both the Tn and CRISPRi libraries in each condition.

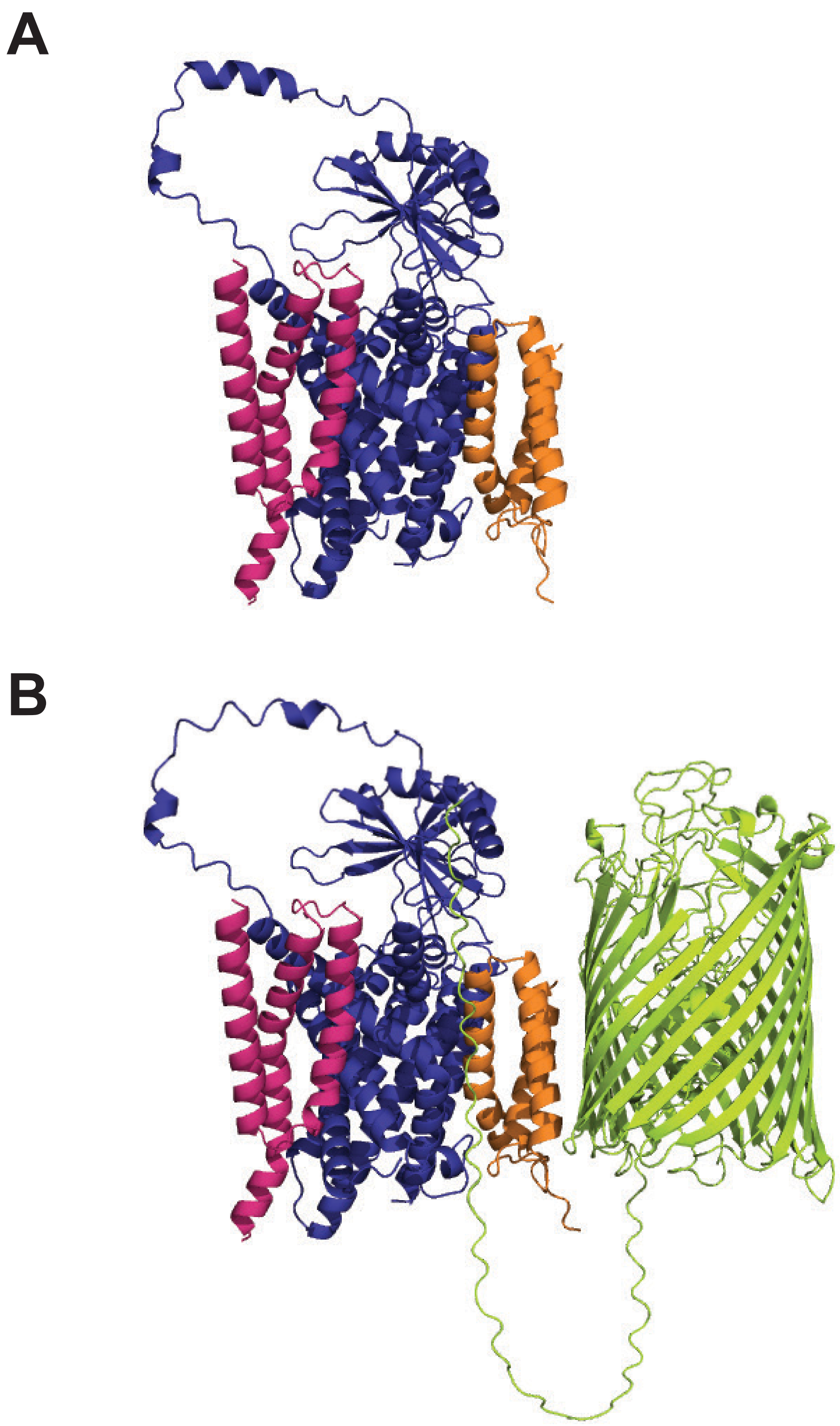

Figure S6. AlphaFold3 predicted complex structure of A) ZMO1628-1630 (iPTM = 0.86) and B) ZMO1628-1631 (iPTM = 0.57). ZMO1628 is blue, ZMO1629 is magenta, ZMO1630 is orange, and ZMO1631 is green.

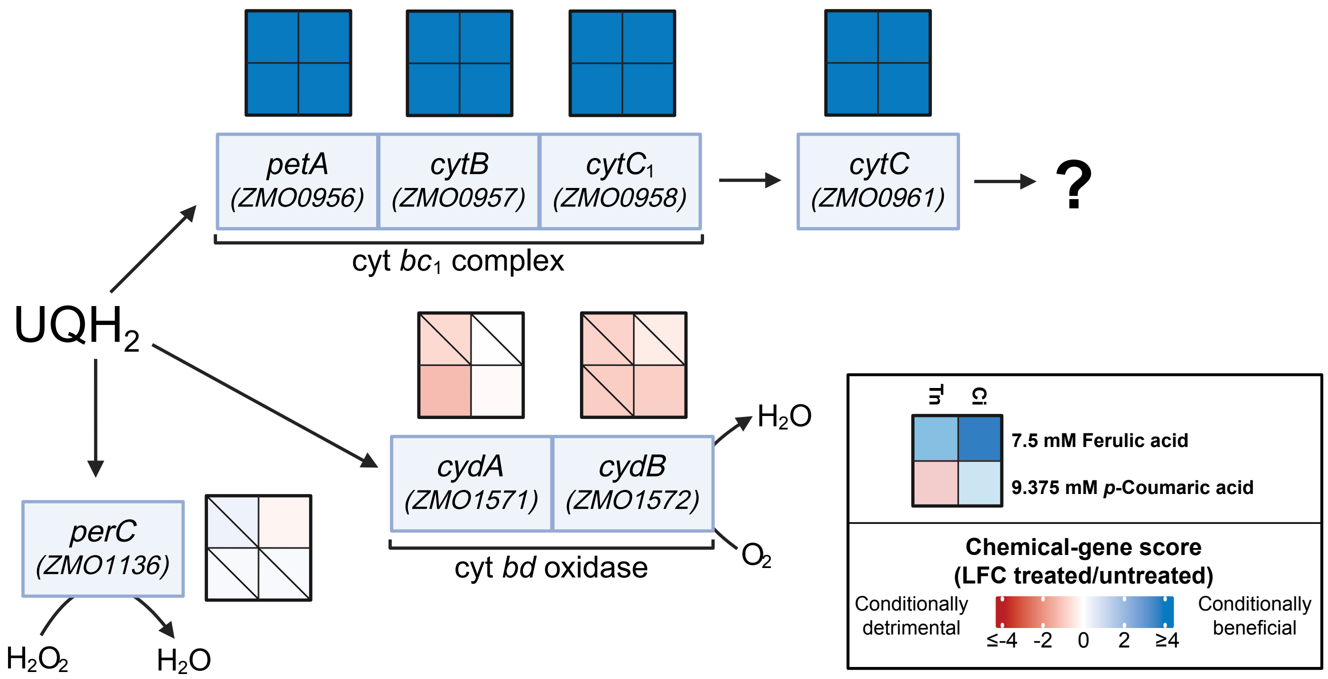

Figure S7. Map of ubiquinol-oxidizing pathways in *Zymomonas mobilis* and their associated chemical-gene scores in ferulic and *p*-coumaric acids. Tn, transposon library; Ci, CRISPRi library. Diagonal lines indicate statistical non-significance (FDR > 0.05).

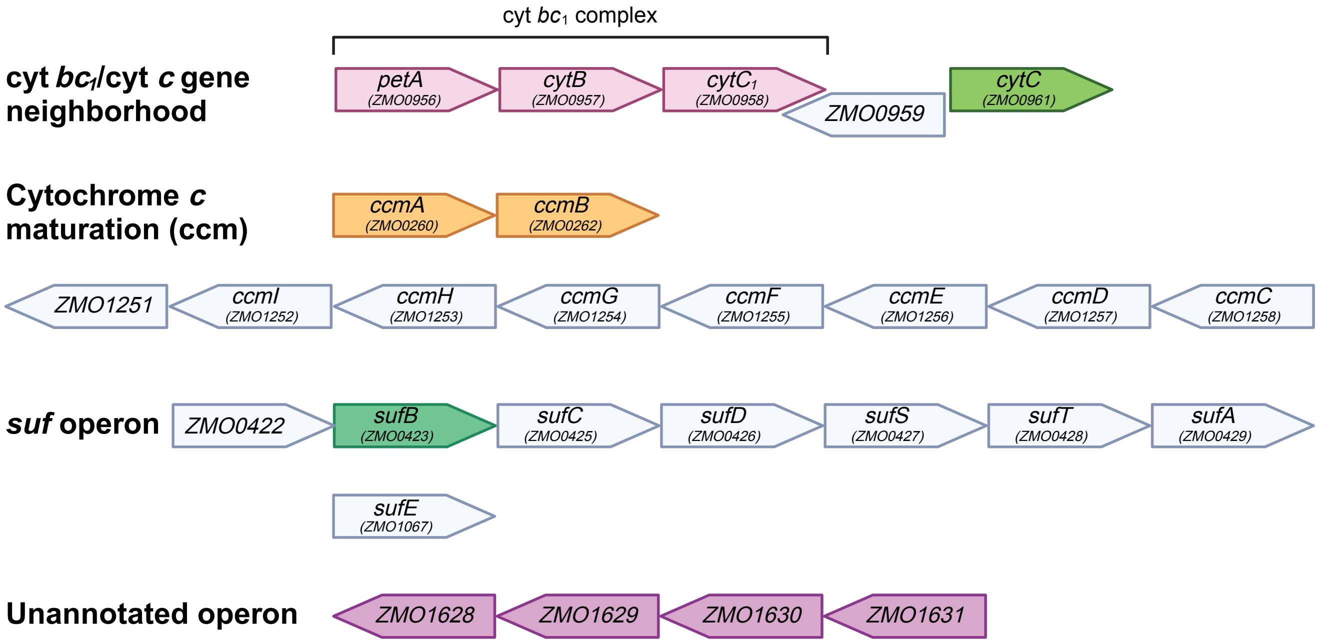

Figure S8. Genomic structure of relevant operons highlighted in our chemical genomics screen. Arrows depicting each gene are not proportional to actual nucleotide length.

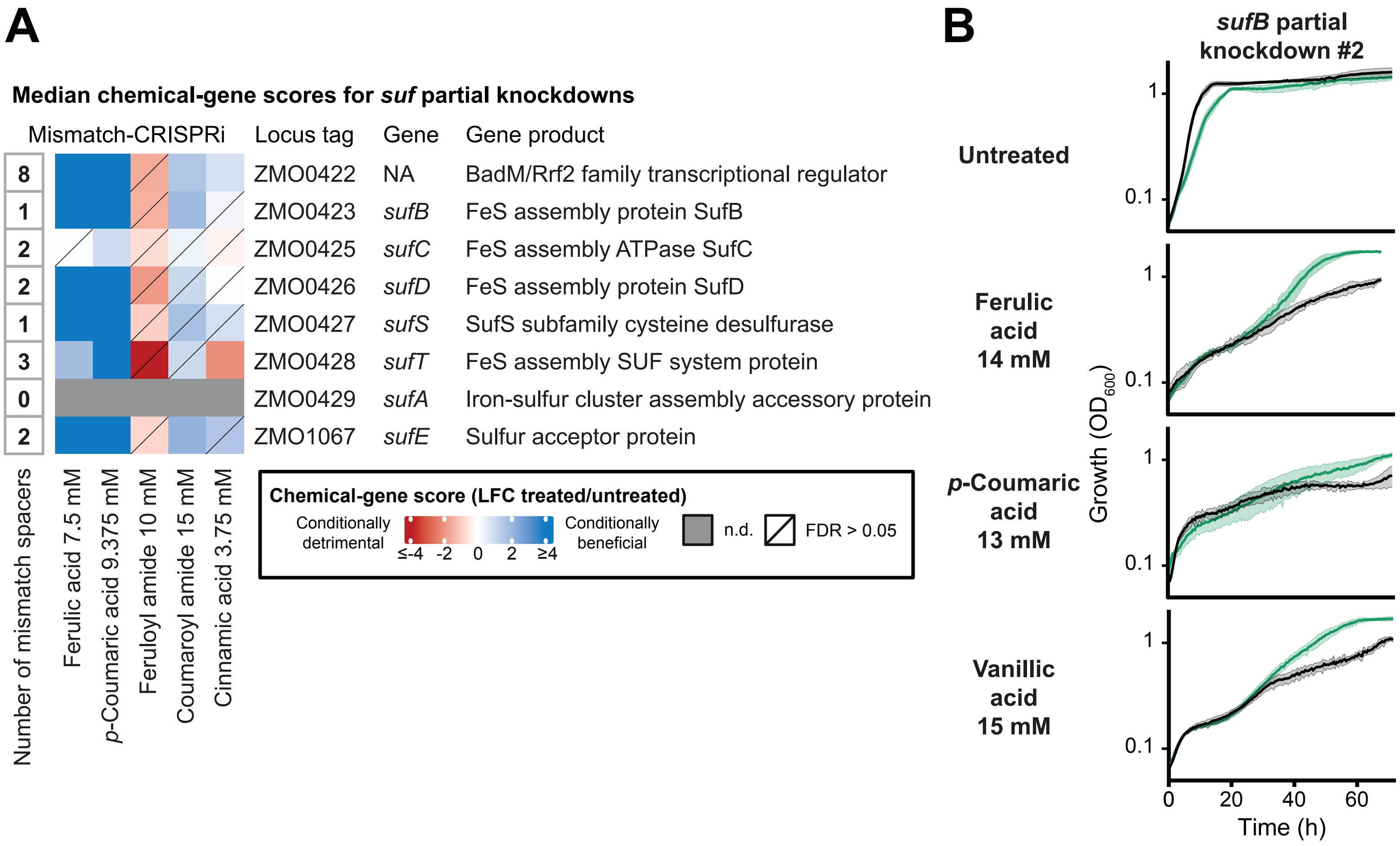

Figure S9. A) Median CRISPRi chemical-gene scores for partial knockdown of *suf* genes. Mismatch sgRNAs in the CRISPRi library that caused a partial fitness defect in the absence of chemical treatment were considered (see Materials and Methods). The number of mismatch sgRNAs analyzed per gene is shown in the left column. B) Anaerobic growth curves of a second *sufB* partial knockdown strain (sJMP7387) with phenolic acids. A CRISPRi non-targeting control is shown in black. Growth curve error bars show standard deviation of quadruplicate samples.

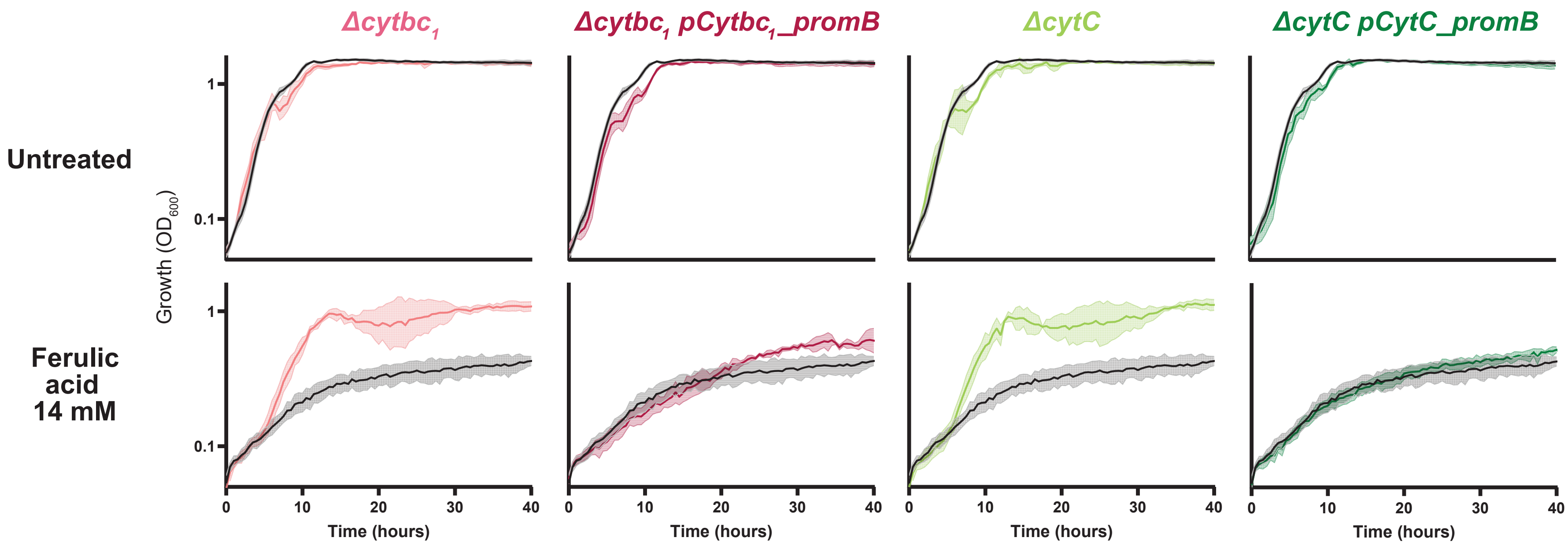

Figure S10. Anaerobic growth curves for *Δcytbc₁* and *ΔcytC* mutants with or without genetic complementation via plasmid-based expression of wild-type genes (colored lines) and wild-type *Z. mobilis* (black lines). Growth curve error bars are standard deviation of quadruplicate samples.

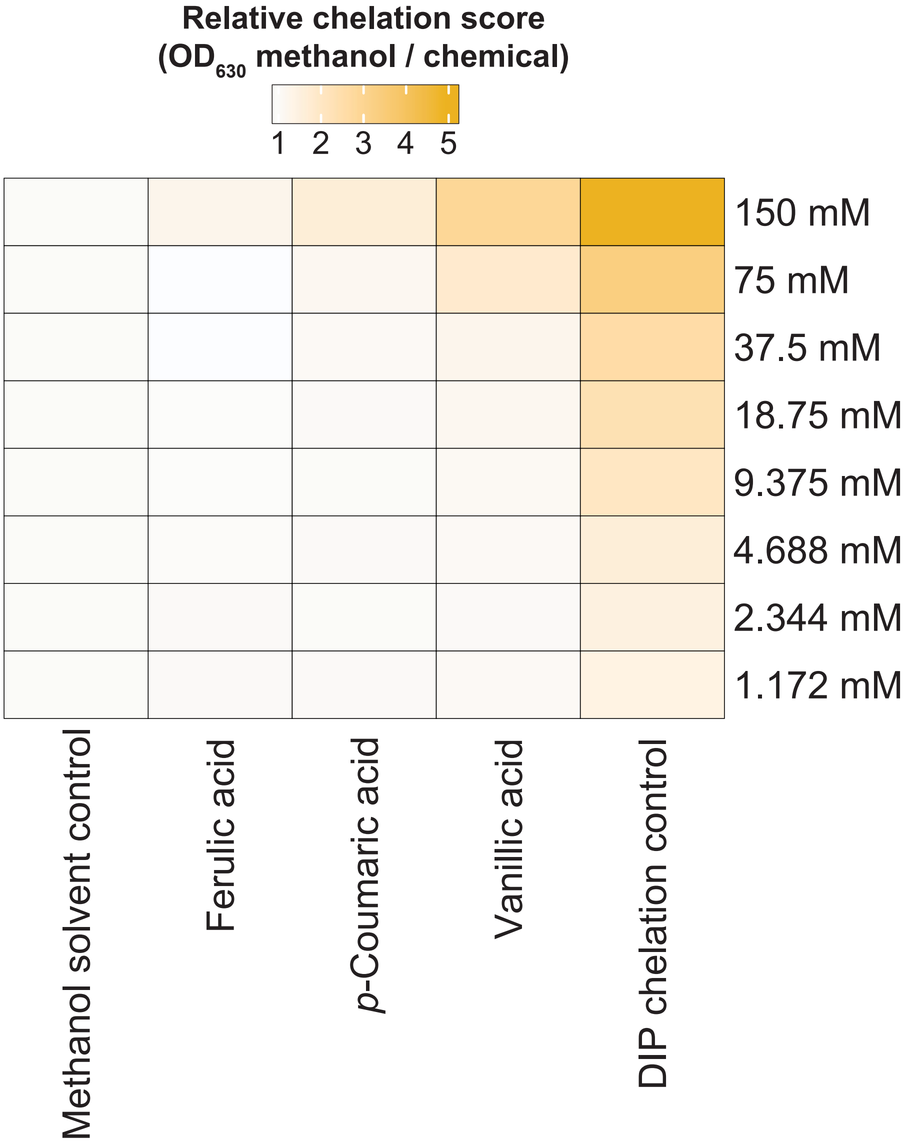

Figure S11. Expanded results of an *in vitro* colorimetric chrome azurol S (CAS) iron binding assay with phenolic acids across a range of concentrations. 2,2 dipiridyl (DIP) is used as a positive control.

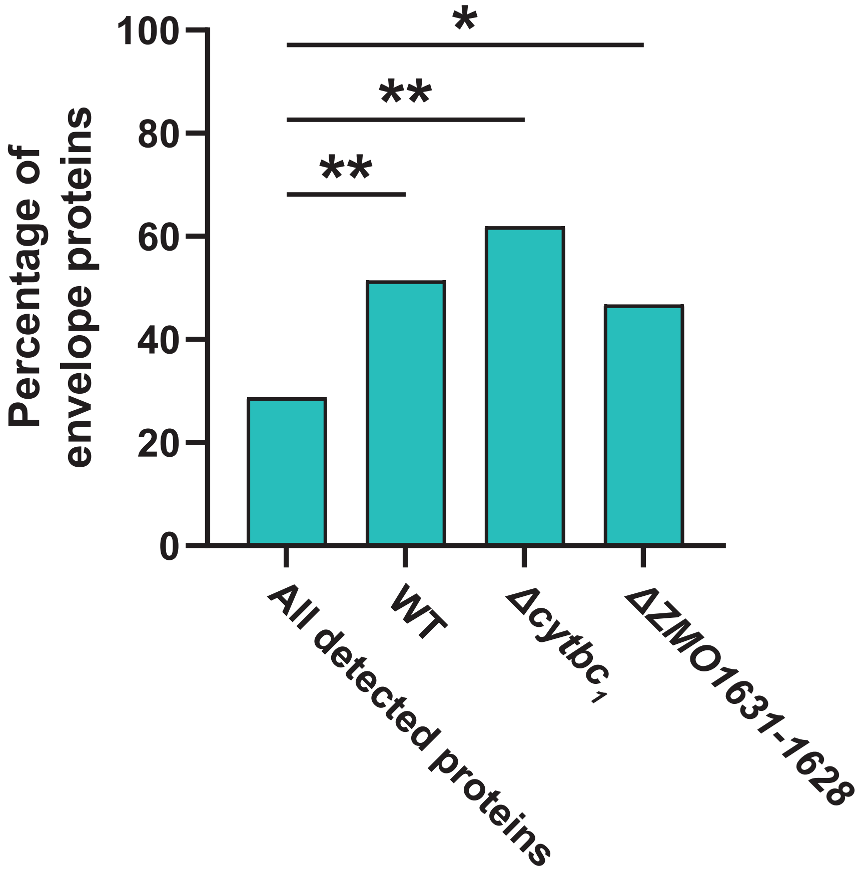

Figure S12. Results of a Fisher Exact Test comparing percentage of envelope-localized proteins between detected proteins and ferulic acid responsive proteins in each strain.

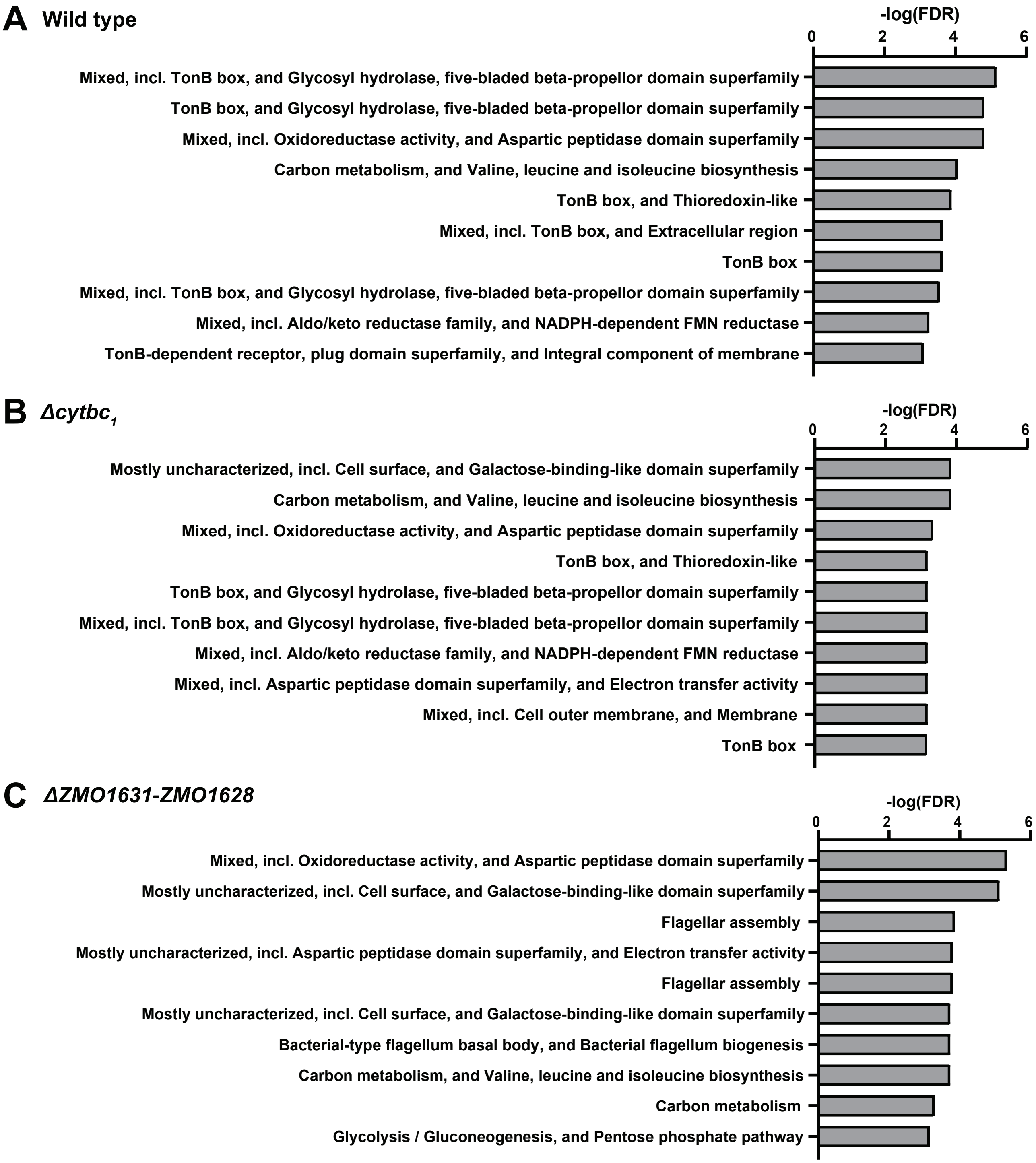

Figure S13. Top ten clusters (sorted by –log(FDR)) from functional enrichment analysis of ferulic acid-treated proteomics data using the STRING database in either A) wild type, B) *Δcytbc*$_1$ or C) *ΔZMO1631-1628*.
